# An optrode array for spatiotemporally-precise large-scale optogenetic stimulation of deep cortical layers in non-human primates

**DOI:** 10.1038/s42003-024-05984-2

**Published:** 2024-03-14

**Authors:** Andrew M. Clark, Alexander Ingold, Christopher F. Reiche, Donald Cundy, Justin L. Balsor, Frederick Federer, Niall McAlinden, Yunzhou Cheng, John D. Rolston, Loren Rieth, Martin D. Dawson, Keith Mathieson, Steve Blair, Alessandra Angelucci

**Affiliations:** 1 Department of Ophthalmology and Visual Science, Moran Eye Institute, University of Utah, Salt Lake City, UT USA; 2 Department of Electrical and Computer Engineering, University of Utah, Salt Lake City, UT USA; 3 SUPA, Institute of Photonics, Department of Physics, University of Strathclyde, Glasgow, UK; 4 Departments of Neurosurgery and Biomedical Engineering, University of Utah, Salt Lake City, UT USA; 5 Mechanical and Aerospace Engineering, West Virginia University, Morgantown, WV USA; 6 Feinstein Institute for Medical Research, Manhasset, NY USA; 7 Present Address: Department of Neurosurgery, Brigham & Women’s Hospital, Harvard Medical School, Boston, MA USA

**Keywords:** Striate cortex, Extracellular recording, Optogenetics

## Abstract

Optogenetics has transformed studies of neural circuit function, but remains challenging to apply to non-human primates (NHPs). A major challenge is delivering intense, spatiotemporally-precise, patterned photostimulation across large volumes in deep tissue. Such stimulation is critical, for example, to modulate selectively deep-layer corticocortical feedback circuits. To address this need, we have developed the Utah Optrode Array (UOA), a 10×10 glass needle waveguide array fabricated atop a novel opaque optical interposer, and bonded to an electrically addressable µLED array. In vivo experiments with the UOA demonstrated large-scale, spatiotemporally precise, activation of deep circuits in NHP cortex. Specifically, the UOA permitted both focal (confined to single layers/columns), and widespread (multiple layers/columns) optogenetic activation of deep layer neurons, as assessed with multi-channel laminar electrode arrays, simply by varying the number of activated µLEDs and/or the irradiance. Thus, the UOA represents a powerful optoelectronic device for targeted manipulation of deep-layer circuits in NHP models.

## Abstract (web summary)

A novel device for selective large-scale optogenetic manipulation of the deep layers of cortical circuits in non-human primates is presented and validated using electrophysiological recordings and c-fos imaging in macaque visual cortex.

## Introduction

Optogenetics has transformed the study of neural circuit function by allowing for the selective modulation of neural activity on a physiologically relevant timescale^1^. Progress in applying optogenetics in non-genetically tractable models, such as the non-human primate (NHP), has lagged behind that in the mouse^2^. Extending optogenetics to NHP studies is crucial, as, due to their similarity to humans, NHPs represent critical models for understanding neural circuit function and dysfunction^3–6^ and provide an essential technology for the development of optogenetic therapies^7,8^. The continuing refinement of viral methods for selectively delivering opsins to particular circuits^9,10^ or cell types^11–13^ is opening up new opportunities to study neural circuits in NHPs^2,14^. Despite these advances, a significant remaining obstacle is the lack of devices for reliably delivering light of sufficient intensity to deep neural tissue across relatively large brain volumes with sufficient spatial resolution to modulate relevant circuit elements.

There are several features of cortical networks that provide both impetus and design requirements for such a device. For example, cortico-cortical feedback connections, which are critical for the contextual modulation of sensory processing^9,15^ and various cognitive phenomena^16,17^, as well as cortico-thalamic feedback projections, arise from deep cortical layers^18,19^. Dissecting these circuits requires selective perturbation of deep layer neurons with high spatiotemporal precision. Moreover, determining whether optogenetic perturbations are limited by the columnar architecture of the NHP cortex, which extends throughout the cortical layers^20^, requires patterned optogenetic perturbations at the spatial scale of cortical columns through the cortical depth. Note, methods for optogenetic stimulation at columnar, or better, resolution recently developed for use in smaller animals^21,22^ only allow for stimulation of the superficial layers in the NHP.

Currently, NHP optogenetic experiments mainly follow two light delivery approaches: through-surface illumination and penetrating probes. Surface photostimulation utilizes either a laser- or LED-coupled optical fiber positioned above the cortex^9^, or chronically-implantable surface LED arrays^23^. These approaches enable photoactivation of a large area, but only to a depth of < 1 mm, due to light attenuation and scattering in tissue. Furthermore, they result in unintended superficial layer neuron activation and even heating damage at the higher intensities required to reach deep layers^9,24^. In contrast, penetrating optical fibers, integrated with single^25,26^ or multiple^27^ recording probes, allow photoactivation at depths >1 mm, but only of a volume a few hundred microns in diameter, and, due to their size and shape, can cause significant superficial layer damage.

To overcome the above limitations, we developed the Utah Optrode Array (UOA), a 10×10 array of glass needle shanks tiling a 4×4 mm^2^ area bonded to an electrically addressable µLED array independently delivering light through each shank^28,29^. Here, we introduce a second-generation device that incorporates a thin, opaque, optical interposer layer between the needle and μLED arrays that increases light coupling efficiency and virtually eliminates stray light. Furthermore, the entire device underwent a robust encapsulation and testing process to enable in vivo testing. Our in vivo testing was performed in macaque primary visual cortex (V1). These experiments demonstrated that the UOA allows for spatiotemporally patterned photostimulation of deep cortical layers with sub-millimeter resolution (i.e., at the scale of single layers and columns) over a large volume. This selectivity can be scaled up to multiple layers and columns by varying the number of simultaneously activated µLEDs and/or the light irradiance. These results establish the UOA as a powerful tool for studying local and large-scale populations of deep-layer neurons in NHP cortex.

## Results

### The UOA: geometry and optical properties

**Fig. 1:**
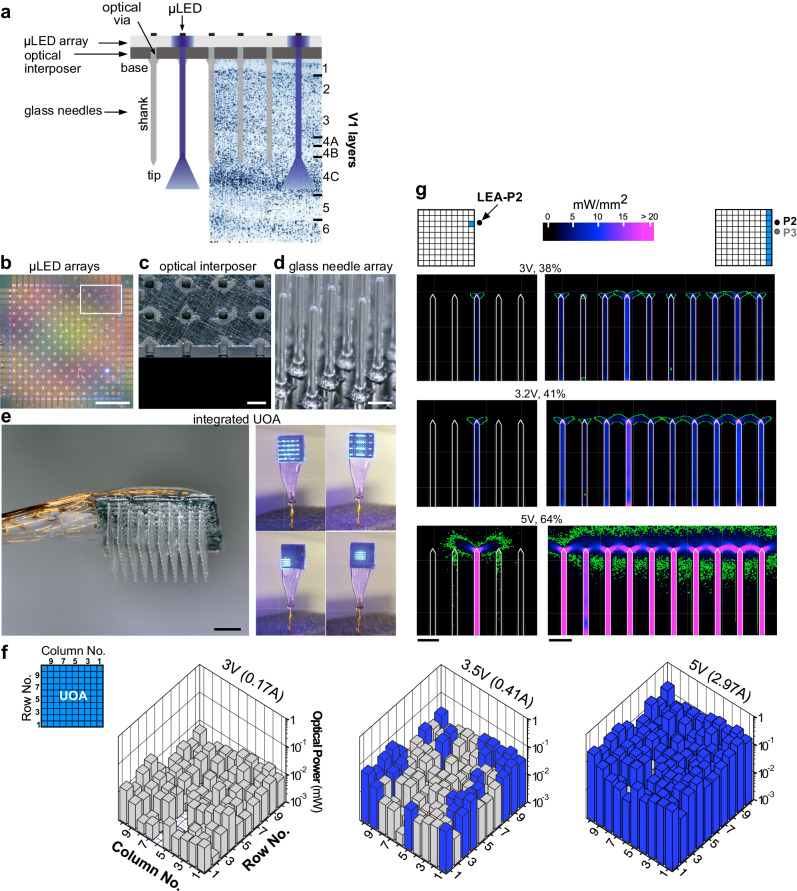
UOA design and optical properties. **a** Schematics of UOA design superimposed to a Nissl-stained coronal section of macaque V1 showing cortical layers. The UOA consists of 3 main components: a µLED array (**b**), an optical interposer (**c**) and a glass needle array (**d**). **b** Two interleaved µLED arrays on a sapphire substrate are shown in this image; the first 10 × 10 array is needle-aligned for deep layer stimulation, the second 9×9 interstitial array lies in-between the first for surface stimulation. The interstitial array, although built into the UOA, was not used in this study. Scale bar: 1 mm. *White box:* location of the region shown at higher magnification in panel **c**. **c** A region of the silicon optical interposer corresponding to approximately the size and location of the *white box* in **b** showing the optical “vias” in correspondence of the needle-aligned µLED array; the optical “vias” are etched through the silicon and matched to the size of a µLED (80 × 80 µm^2^). Scale bar: 200 µm. **d** High magnification image of the glass needle shanks bonded to the interposer. Scale bar: 200 µm. **e** Left: the µLED on sapphire and needle array components are integrated into the final device, wire-bonded, and encapsulated. The image shown is a representative device. The integrated UOA used in this study consisted of 10×10 glass needle shanks, 1.6 mm long (to target deeper layers) and 100–110 µm wide, with tip apex angles of ~64°. An image of the actual device used in the in vivo testing studies, after completion of the experiment and explanation is shown in Supplementary Fig. 3. Scale bar: 1 mm. Right: Example spatial patterns of device operation. **f** Average output optical power (in mW) across each needle tip at different drive voltages (currents), when the entire UOA was turned on (top left inset). Blue and gray bars: needle shanks with estimated tip irradiances above and below, respectively, the 1 mW/mm^2^ threshold for ChR2 activation. **g** Left: Ray trace model (see “Methods” section) of light spread in cortical tissue when a single µLED (in column 1 and row 8, i.e., the closest to the linear electrode array —LEA– in penetration 2 –LEA-P2– used for the electrophysiological testing experiment, and indicated as a black dot in the UOA cartoon) is activated at various input voltages (% of maximum intensity used- top to bottom rows represent driving voltages of 3 V, 3.2 V and 5 V), with power output calibrated to the bench tests. Right: Model of light spread in tissue when all of column 1 (the nearest to the LEA in LEA-P2 and LEA-P3) is activated at various input voltages (rows). *Green contours* in all panels enclose tissue volume within which the light irradiance is above 1 mW/mm^2^, the threshold for ChR2 activation. Scale bars: 400 µm.

The UOA is based on the geometry of the Utah Electrode Array (UEA)^30^. Similar to the UEA, it is a 10 × 10 array of penetrating glass optical light guides (needles), with customizable length (up to 2.5 mm) and shank width (80-120 µm) on a 400 µm pitch, tiling 16mm^2^. Shank width (80–120 µm) is smaller than that of the UEA (150 µm) at the base, and unlike the UEA does not taper (as this would introduce light leakage). The tip geometry of the UOA can be controlled through the fabrication process, but the tips are generally slightly wider than those of the UEA. A custom μLED array fabricated on a GaN (gallium nitride) on Sapphire wafer is directly integrated with the device, with each electrically addressable 80 × 80 µm μLED delivering 450 nm light through a single needle (Fig. 1a–e). A second 9×9 array of “interstitial” µLEDs is interleaved on the same device for independent surface stimulation (as shown in Fig. 1b, but not used in this study). To limit the spatial spread of coupled light, the first generation UOA used a metal pinhole array^28^. Bench testing demonstrated the potential of this device for delivering patterned light at irradiances in excess of activation thresholds across a range of commonly employed depolarizing^31^ and hyperpolarizing^32^ opsins, with a 50% decrease in irradiance within tissue approximately 200 µm from a needle tip^28^, thus providing for depth selectivity. These initial results suggested that direct optogenetic activation through the UOA is on a spatial scale commensurate with the functional architecture of primate cortex.

Here we have developed the second-generation UOA, which incorporates an optically opaque interposer layer with circular openings (optical “vias”) through which light emitted by the µLEDs only transmits to the optrode shanks, which eliminates unwanted surface illumination and inter-needle crosstalk (Fig. 1a, c and Supplementary Fig. 1; see “Methods” section for manufacturing details), and, with the high thermal conductivity of Si, acts as a heat spreader to reduce hot spots during operation. This device (Fig. 1a–e) was first bench tested (Fig. 1f), and from those measurements, in vivo optical performance was estimated via ray tracing (Fig. 1g; see “Methods” section). Maps of output power, at each needle tip at different drive voltages, are shown in Fig. 1f (Supplementary Fig. 2 also shows the estimated output irradiances). At 3 V, output power and estimated irradiance levels are below the 1 mW/mm^2^ threshold for the excitatory opsin *Channelrhodopsin-2* (ChR2) (Supplementary Fig. 2 and Supplementary Table 1). Note that defining the irradiance emitted from faceted optrode tips is challenging. For simplicity, in Supplementary Fig. 2b, we define the irradiance as the emitted optical power divided by the area of the emission surface; however, optical modeling indicates that the emission is non-uniform across the tip surface, with higher irradiance near the tip apex (Fig. 1g). There is also variation in emission across the array, due primarily to variations in the resistance (and therefore slope efficiency) of each μLED. At 3.5 V, ~30% of the stimulation sites reach or exceed ChR2 threshold (mean optical power ± SD = 0.022 ± 0.013 mW; mean irradiance = 0.82 ± 0.49 mW/mm^2^), while at 5 V, more than 90% of the sites emit above threshold (0.1 ± 0.056 mW; 3.79 ± 2.08 mW/mm^2^). In principle, software modifications can be made to the Arduino microcontroller on the matrix driver board to better equalize stimulation levels across the array.

Using optical ray tracing, we estimated the direct neural stimulation volume (based upon the local irradiance in tissue) as a function of drive voltage and pattern of activated needles to facilitate interpretation of the in vivo results (see “Methods” section). The left column panels in Fig. 1g show the stimulation volume, in cross-section, along the first UOA column as produced by the needle (column 1, row 8) nearest one of the electrode penetrations (penetration 2 –LEA-P2) in the in vivo experiments; the right column panels show the activation volume when all of column 1 is activated. Different rows depict activation volume for different drive voltage levels (from top-to-bottom row 3–5 V, respectively). At low drive voltage (~3 V; top row in Fig. 1g), highly localized stimulation in tissue near the needle tips is produced (as mentioned, optical modeling indicated the irradiance across the tip surface is non-uniform – concentrated near the apex – explaining why above-ChR2-threshold irradiance levels can be achieved at 3 V). At higher voltages (≥5 V; bottom row in Fig. 1g), the stimulation volume overlaps that of adjacent needles, while also extending deeper into tissue. When driving an entire column, at 3 V (Fig. 1g top right panel), stimulation localized near each tip is mostly retained, whereas a nearly continuous stimulation volume is obtained at 3.2 V (Fig. 1g middle right panel) due to overlapping intensity patterns. At 5 V (Fig. 1g bottom right panel), the depth of this continuous volume increases, both above and below the tips.

### In vivo testing: electrophysiology

**Fig. 2:**
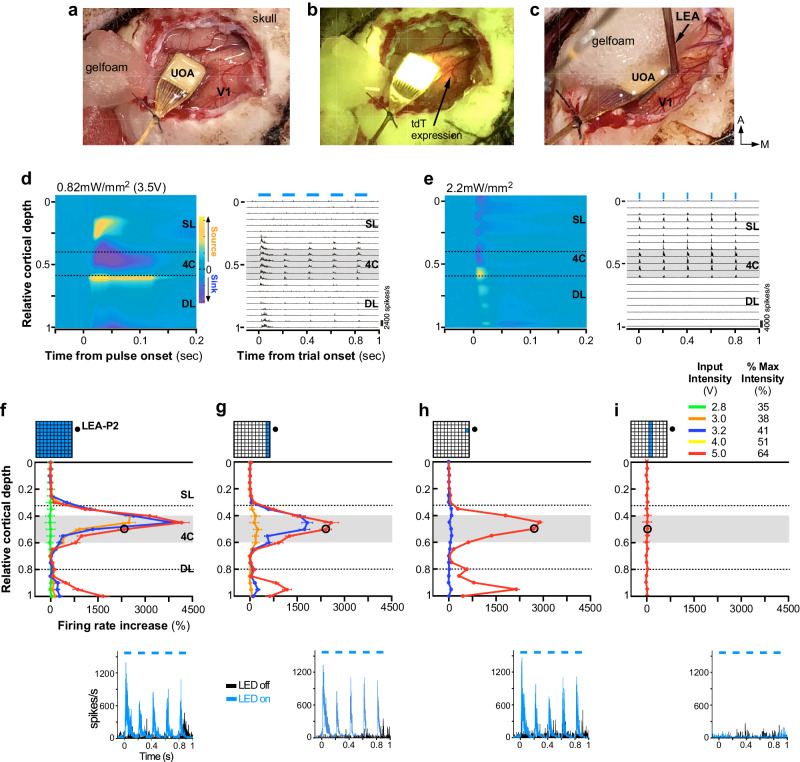
Laminar distribution of responses induced by UOA photostimulation. **a** The UOA used in the in vivo experiments inserted in macaque V1. **b** Same field of view as in (**a**) shown under fluorescent illumination to reveal expression of the red fluorescent protein tdTomato (arrow). **c** Preparation for recording electrophysiological responses to photostimulation. A 24 channel linear electrode array (LEA) was inserted next to the UOA (guide tube protecting array marked “LEA”) slightly angled laterally (towards the UOA) and posteriorly. Here the UOA is partially covered with a piece of Gelfoam. d Current Source Density analysis (CSD; Left) and MUA (Right) signals recorded through the depth of V1 in LEA-P2 in response to phasic UOA photostimulation (pulse parameters: 100  ms pulse duration, 5 Hz, 0.82  mW/mm^2^; pulse periods denoted as blue bars above MUA plot). Here, all 100 needle-aligned µLED sites (“whole µLED array” condition) were activated simultaneously. CSD responses to each 100  ms pulse were zero-aligned (*n* = 170 pulses), while MUA is shown for the full 5  Hz pulse train (*n* = 34 trials). The dashed lines in the CSD panel demarcate the borders of layer 4 C (L4C); the gray shaded region in the MUA panel delimits the extent of L4C. **e** Same as in **d**, but following surface photostimulation of V1 via a laser-coupled optical fiber with pulse parameters of 10 ms, 5 Hz, 2.2  mW/mm^2^ (*n* = 160 pulses, 32 trials). **f**–**i** Top: relative cortical depth of each contact on LEA-P2 (black dot in the insets) is plotted versus the relative response (% firing rate increase over baseline) to UOA stimulation for different 450  nm µLED illumination patterns (top insets) (*n* = 125–205 pulses per condition). Different colored traces are data for different photostimulation intensities (expressed as voltage or percent of max intensity used). Gray area: extent of L4C; dashed lines: approximate location of the L4A/4B (upper) and L5/L6 (lower) borders. In all panels, error bars represent standard error of the mean. Bottom: PSTHs with and without µLED activation are shown for the same contact on the LEA in L4C (marked by the black circle in the graphs above) across conditions. Dashed line in the PSTH: pulse periods. In all panels, error bars represent standard error of the mean.

We used in vivo linear electrode array (LEA) recordings to determine whether stimulation through the UOA could modulate selectively deep layer neurons expressing ChR2. ChR2 and tdTomato (tdT) were expressed in macaque V1 via a mixture of Cre-expressing and Cre-dependent adeno-associated viral vectors (AAV9)^9^. Following a post-injection survival period of 9 weeks, to allow for sufficient expression of ChR2, we recorded multi-unit spiking activity (MUA) in acute experiments in anesthetized macaque using a 24-contact LEA inserted nearby a UOA implanted into a region of dense tdT expression (tdT fluorescence was imaged in vivo prior to UOA implantation to guide placement) (Fig. 2a–c and Supplementary Figs. 3 and 4a). We performed three LEA penetrations (LEA-P1-P3), but modulation of neural activity via UOA photostimulation was only detected for LEA-P2 and LEA-P3 (likely because P1 was farthest from the region of tdT/ChR2 expression; see Supplementary Fig. 4a and Discussion). Below we report data from LEA-P2 and LEA-P3.

### Comparison of surface and UOA photostimulation

Figure 2d shows neural activity recorded in response to simultaneous activation of µLEDs at all UOA sites (LEA-P2 - whole array condition) at an irradiance level of 0.82 ± 0.49 mW/mm^2^ (pixel-pixel average ± SD), roughly equivalent to ChR2 activation threshold^31^, induced by an input intensity of 3.5 V (see Supplementary Table 1 for irradiance values at each site). To examine the effect of UOA stimulation on activity across all V1 layers, we first performed a current source density (CSD) analysis of the local field potential (LFP) recorded across all LEA contacts around the time of the first pulse in a trial (to avoid effects due to preceding pulses) (see Methods). No visual stimuli were presented during UOA stimulation. The CSD reveals the location of current sinks (negative voltage deflections reflecting neuronal depolarization) and sources (positive voltage deflections reflecting return currents) throughout the cortical depth. We used spiking responses to identify the top and bottom of cortex, normalizing cortical depth by assigning to these values of 0 and 1, respectively, and then identified layer (L) 4 C on the basis of post-mortem histology – see Methods). Current sinks following UOA stimulation were mostly localized to L4C and the lower part of the deep layers. Similarly, we observed strong phasic multi-unit activity (MUA) following UOA stimulation that was also confined to L4 and the lower part of the deep layers, with L4C activity preceding deep layer activation (Fig. 2d). This suggests that the UOA needle tips closest to LEA-P2 terminated in L4C (later confirmed by further experiments and post-mortem histology, see below) and that, at these low photostimulation intensities, light spread remained close to the UOA tips. In contrast, at the highest drive intensity we tested (7.8 V), consistent with the optical ray tracing modeling described above and in Supplementary Fig. 5a, the laminar pattern of V1 activation suggested that light spread farther into deeper layers (Supplementary Fig. 5a, b). Importantly, at this drive intensity (7.8 V), neural activation could not be explained by thermal artifacts (Supplementary Figs. 6 and 7).

Comparison of the laminar activity patterns elicited by UOA photostimulation with that elicited by direct surface photostimulation in a different animal at a slightly higher irradiance (2.2 mW/mm^2^) revealed a sharp dissociation. Specifically, surface stimulation of ChR2 evoked responses starting in the superficial layers and terminating in L4C **(**Fig. 2e).

Finally, to determine which UOA needles were closest to a particular LEA penetration, we analyzed the effect of varying UOA stimulation at single sites along column 1 (the closest column to all LEA penetrations). This analysis revealed that the UOA needles closest to LEA-P2 were those in rows 8 and 9 (labeled C1-R8, and C1-R9, respectively – Supplementary Fig. 4b, Left. Additional analysis of this dataset demonstrated that response onset latency and onset reliability were lowest and highest, respectively, for the LEA-P2 contacts located in L4C. Combined with postmortem histological assessment, this confirms the UOA needle tips closest to LEA-P2 were located in L4C (Supplementary Fig. 4a, b, Right). Applying this same analysis to data from LEA-P3 also allowed us to determine the location of this penetration relative to the UOA. In this case, site C1-R7 was closest to the LEA, and its tip terminated in the superficial layers (Supplementary Fig. 4a, c).

### UOA stimulation parameters can be tuned to achieve laminar specificity

To assess the impact of UOA stimulation on MUA we varied: (i) the spatial pattern of UOA stimulation (single μLED sites, entire columns, or the entire device), and (ii) stimulation intensity across these spatial patterns. In all conditions, we used phasic stimulation (5 Hz, 100 msec pulses for 1 sec with 1.5-21 sec inter-trial intervals, with the longer intervals used at the higher stimulation intensities) with a slow on/off ramping to eliminate the potential of any electrical artifacts induced by capacitive coupling at the array/tissue interface^33^. As an example, Fig. 2f–i shows responses from LEA-P2. As indicated by an analysis of firing rate increase across layers induced by activating a single µLED at different sites along column 1, the UOA needles closest to LEA-P2 were those in rows 8 and 9 (C1-R8, C1-R9), and their tips terminated into L4C (Supplementary Fig. 4b Left). The laminar distribution of MUA in LEA-P2 varied in amplitude across conditions, but was reliably confined to deeper layers. By varying the spatial pattern of stimulation and/or the stimulation intensity, MUA could be confined to single layers or spread across multiple layers. For example, activation of the whole UOA (Fig. 2f) at input intensities between 2.8-3.2 V evoked a MUA peak within L4C (again, where the needle tips nearest to LEA-P2 terminated). This peak increased in magnitude with increasing stimulation intensity. Further increases in input intensity (4–5 V) led to a second, smaller, MUA peak in L6 (but not L5). In macaque V1, L4C projects to both L5 and L6^34^, but its net effect is to suppress L5^35^ and excite L6^36^. This suggests that, at our higher stimulation intensities, L6 responses (without concomitant L5 activation) may have resulted from monosynaptic spread from optogenetically activated L4C neurons. Similarly, the limited superficial activation we observed across conditions could have been due to L4C to either L4B and/or L3 connections. While we did not observe consistent suppression below baseline activity for MUA in L5 during UOA activation, this could have been due to our analysis of the MUA rather than single-unit signals, or simply to a lack of sensitivity due to low baseline firing rates. Below, we provide evidence further supporting this circuit interpretation.

Finally, for the whole array condition, at the highest input intensity, neural activation extended from L4C through L6, likely via direct activation of the deeper layers due to light scattering through a larger volume (Supplementary Fig. 5c, Left). Previous work has demonstrated modulation of spiking activity when local temperature changes exceed 1–2°C^9,24^. We performed a finite element model analysis of the thermal performance of the UOA and found that in all conditions, save stimulation through the entire UOA at the highest intensity, peak temperature change in cortex remained below 1 °C during the 1 s trial period (Supplementary Fig. 6). However, under certain conditions, peak temperature could continue to rise with successive pulses in a train, remaining below threshold for heat-induced spiking modulation but failing to relax to baseline by the end of the trial period. Thus, there is potential for a cascading effect, whereby tissue temperature at the beginning of each subsequent trial is slightly higher than the previous. This could lead, after a series of trials, to a greater than 1–2 °C change in temperature with consequent effects on spiking activity. To guard against this possibility, during conditions in which we activated a number of µLEDs at higher intensities, we used longer inter-trial periods (up to 20 s), which we hypothesized would allow tissue temperatures to return to baseline levels by the start of the next trial.

We reasoned that, if we were in fact raising cortical temperatures more than 1–2 °C, and this temperature change persisted after µLEDs were turned off, we would see changes in neuronal activity during the inter-trial period. To quantify this effect, we chose to compare firing rates during three portions of the ITI (of equivalent duration), early, middle, and late, to resting firing rates recorded before we began an experiment. We reasoned that comparing the pre-experiment activity, when the network is in a resting state, with the inter-trial period would be a reasonable basis for detecting differences in baseline activity (i.e. spiking in the absence of any visual or optogenetic stimulus). If ITI period activity was significantly different from the pre-experiment baseline, then heat might have caused a change in the neurons’ resting period activity. Notably, we did not observe consistent changes in ITI spiking, relative to pre-experiment baseline, by the end of the ITI across all conditions (Supplementary Fig. 7). This suggests that even if we did induce thermal artifacts, they had dissipated by the beginning of each trial.

Although thermal artifacts could not explain the findings at the highest intensity tested with our stimulation parameters, in general, lower stimulation intensities should be favored in experiments, particularly when the entire UOA is activated and shorter inter-trial intervals are used.

Activation at 5 V evoked similar laminar patterns and magnitudes of MUA irrespective of whether a single µLED, an entire column nearest the LEA, or the whole UOA were illuminated (Fig. 2f–h). However, at lower intensities, firing rate increased with the number of activated µLEDs (e.g., compare blue curves in Fig. 2f–h). In this range, an input intensity exceeding 3.2 V was required to modulate neural activity via stimulation through a single µLED (Fig. 2h). When stimulating through an entire UOA column, moving the activation column by a distance of 1.6 mm on the UOA (from column 1 to 5) resulted in a 10-fold reduction in MUA amplitude (Fig. 2i). At this farthest site tested, increases in L4C activity were observed only at the highest input intensity (7.8 V; Supplementary Fig. 5c, Right). No increase in firing rate could be evoked by activation of an entire column beyond this distance or by activation of a single µLED in column 1 beyond a similar distance on the UOA (C1-R4; corresponding to a distance from the LEA of 2.6–2.7 mm as estimated from postmortem histology) even at the highest input intensity (again, 7.8 V, Supplementary Table 1).

### Tangential extent of responses induced by photostimulation via the UOA

**Fig. 3:**
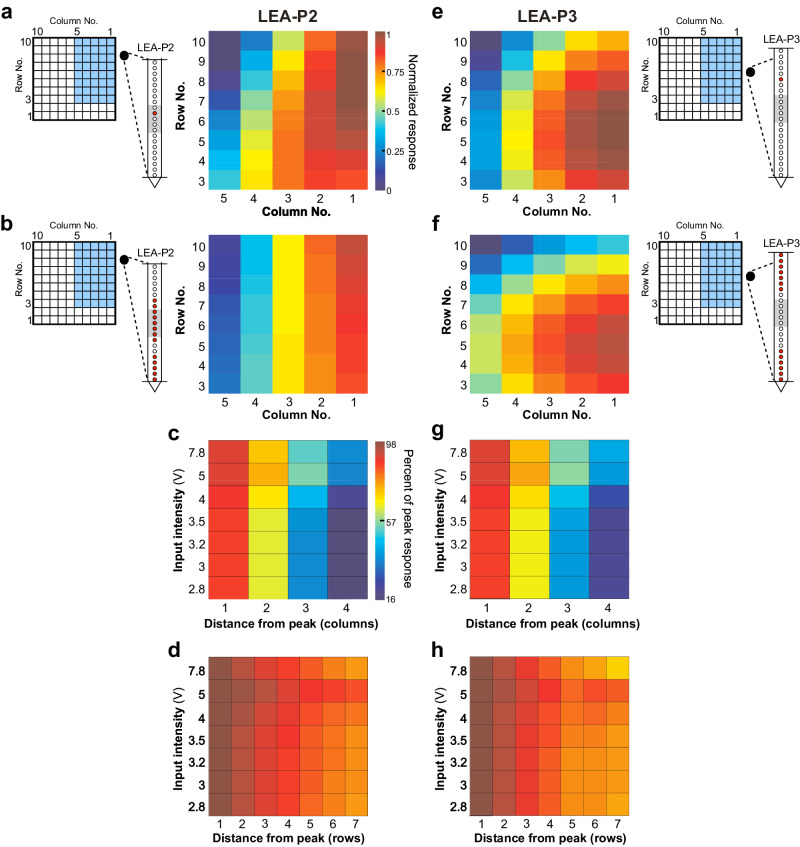
Tangential extent of responses induced by UOA photostimulation. **a** Examples of model fits to single μLED photostimulation for the contact from LEA-P2 showing the largest relative response increase across these stimulation conditions. This contact preferred stimulation in the proximal UOA columns 1–2, at sites closer to the top of the device (rows 9–7). The schematics on the left of the UOA and of the LEA-P2 indicates as blue shading the UOA sites represented in the heat map, and as a *red dot* the contact on the LEA whose response is mapped on the right. The horizontal lines and gray shading on the LEA schematics mark the pial and white matter, and L4C boundaries, respectively. Color scale applies to panels (**a**, **b**, **e**, **f**). **b** Average normalized fitted responses across all responsive contacts in LEA-P2 (*red dots* in schematics of LEA to the left). **c** Change in response in the column direction for LEA-P2. Average relative response amplitude (percentage of peak model-fitted response) is plotted as a function of stimulation intensity and distance along a straight line extending from the preferred UOA site in the column direction, and sorted by input intensity. Data averaged across all responsive contacts. **d** Change in response in the row direction for LEA-P2. Average relative response amplitude (percentage of peak response) is plotted as a function of stimulation intensity and distance along a straight line extending from the preferred UOA site in the row direction. Data averaged across all contacts. **e**–**h** Same as in **a**–**d** but for LEA-P3.

We next investigated whether the MUA at different cortical sites was selective for the spatial locus of UOA stimulation. That is, did stimulation at different UOA locations result in greater or lesser activation of neurons near our LEA contacts, and, if so, did this selectivity for the site of UOA stimulation vary across the cortical depth? To estimate MUA selectivity for stimulation at UOA sites between columns 1–5 and rows 3–10, we fit a multiple linear regression model to the MUA recorded at each LEA contact, with row, column, and intensity (V) as independent variables (see Methods). We included here only contacts on which there was a significant difference in firing rates during the stimulation and control periods for at least one of the row or column conditions (ANOVA, all *p* < 0.01). Across the population, including a quadratic term explained more of the variance in the MUA response (mean *R*^2^ ± SD: 0.58 ± 0.14 vs. 0.31 ± 0.11 for a linear model; Kolmogorov-Smirnov test for differences in the distribution of R^2^ values across all linear and quadratic model fits, *p* < 10^−7^). Figure 3a,e shows plots of fitted MUA for 3.5 V single-µLED photostimulation for the contact in LEA-P2 and LEA-P3, respectively, which showed the greatest relative response modulation. For each contact, we normalized responses to the peak (e.g., generating maps like those shown in Fig. 3a, e), and averaged across contacts to determine whether MUA recorded over different LEA penetrations preferred stimulation at different UOA sites (Fig. 3b, f). Consistent with our prior assessment (Supplementary Fig. 4a–c) of LEA placement relative to the UOA, peak locations differed significantly across the two penetrations (ANOVA, *p* < 0.01). Specifically, the peaks for LEA-P2 contacts tended to cluster mostly near C1-2/R8-9, while those for LEA-P3 contacts clustered mostly near C1-3/R4-7. The spatial distribution of preferred UOA stimulation sites across LEA contacts suggested that, particularly for LEA-P3, the LEA was inserted at a slightly oblique angle relative to the cortical surface.

The data in Fig. 2g, i and Fig. 3a, b, e, f indicated MUA amplitude decreased with increasing distance between photostimulation and recording sites. To quantify this observation and better characterize the extent of photostimulation-evoked responses across the tangential domain of the cortex, we examined MUA amplitude as a function of distance on the UOA (in a straight line extending along either the row or the column axis) from the site that evoked the peak response (Fig. 3c, d, g, h). As is evident from the steeper decrease in responses along the column versus the row axis, as well as the difference in relative response across stimulus intensities, there was a significant effect of UOA axis and input intensity on relative response (ANOVA, both *p* < 10^−21^), as well as a significant difference across penetrations (ANOVA, *p* < 10^−14^). Finally, there was a significant interaction between intensity and UOA axis as well as UOA axis and penetration (ANOVA, both *p* < 0.01). These results indicate that the response decrease from peak is greater in the column versus the row direction, that intensity has a different effect on this drop-off in the row versus column directions, and that this differed across penetrations. For example, in the column direction, at an input intensity of 2.8 V, MUA dropped to 16% of peak at a distance of 1.6 mm from the site eliciting peak activation. Conversely, at ≥ 5 V it dropped to only 50% of the peak response at the same distance (Fig. 3c, g). This is in sharp contrast to the row direction, where, at an input intensity of 2.8 V MUA only dropped to 80% of peak at a distance of 2.8 mm (90% at ≥5 V) (Fig. 3d, h). This difference in the magnitude of response decrease with distance in the column vs. row directions is likely explained by the greater differences in irradiance, for a given input intensity, along the column as compared to the row axis (see Supplementary Fig. 2).

In summary, the spatial spread of MUA along the tangential domain of cortex varied according to UOA stimulation site and intensity. Importantly, the extent of this spread was more limited at lower intensities, suggesting that increasing intensity increased the volume over which cells were optogenetically activated, consistent with the model simulations shown in Fig. 1g. Note also the potential for spatial resolution of UOA stimulation to vary with factors intrinsic to the cortical area under study (e.g., the extent of intra- and inter-laminar connectivity) as well as with experimental factors (device implantation, transgene expression levels, etc.).

### UOA activation parameters can be tuned to activate distinct cortical networks

Given the spatial separation between the LEA and the UOA (~1–1.1 mm for LEA-P2 and 700–800 µm for LEA-P3, based on histology; Supplementary Fig. 4a), the reported sharp falloff in light intensity over short distances in tissue^37,38^, and our bench estimates of light spread from the UOA tips^28^ (see also Fig. 1g), we reasoned that the evoked MUA we recorded was likely relayed to the recorded neurons indirectly, via activation of ChR2-expressing cells nearby UOA needle tips. To examine this possibility, we measured the onset latency of evoked MUA across layers.

**Fig. 4:**
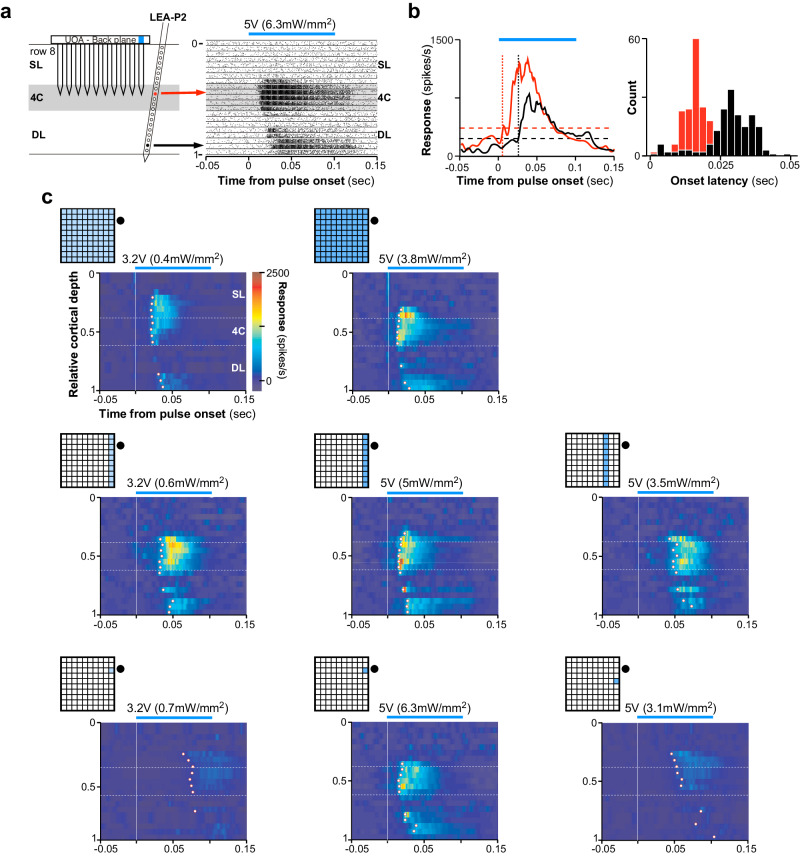
Onset latencies reveal local networks activated by focal optogenetic modulation. **a** Left: schematics of UOA stimulation (*n* = 175 pulses) through a single μLED site (C1-R8) and of relative LEA position in LEA-P2. Right: Pulse-aligned raster plots for all 21 channels on the LEA through the depth of V1. *Black lines* separate data from different channels*. Gray shaded region:* channels in L4C. *Blue line above plot*: 100 ms pulse period at the input voltage (irradiance) indicated*. Red and black arrows denote example contacts in L4C and 6, respectively*. A graded shift in MUA onset latency is apparent. (**b**) Left: Pulse-aligned PSTHs for the two channels indicated by arrows in the raster plot in (a). Responses are plotted as baseline-subtracted firing rate versus time. Response onset latency at the L6 contact (35 ms) clearly lagged that on the L4C contact nearest the UOA needle tips (17 ms). Right: Histograms of pulse-by-pulse onset latencies for the two example contacts. (**c**) Heatmaps of MUA (firing rate) through the depth of V1 during the peri-pulse period (*n* = 125 – 205 pulses per condition), for the UOA stimulation condition indicated by the insets at the top left of each plot. Stimulation intensity (average irradiance) is reported above each plot. The firing rate color scale applies to all panels. *White dots* mark the onset latency (estimated from the mean PSTH – see Methods) for each contact that was significantly responsive to UOA stimulation.

Example latency data from LEA-P2 are shown in Fig. 4a. Here, the UOA stimulus was a single μLED (C1-R8/5 V) nearest the recording location. The fastest evoked response occurred in L4C with an onset latency (see “Methods” section) of ~15 ms. Note that the onset kinetics of the ChR2 variant we expressed in V1 (see ”Methods” section) has been estimated on the order of 2 ms^39^. Thus, even taking into consideration that we employed a criterion measure of latency that may have overestimated the time at which the first optogenetically driven spikes were recorded, the evoked responses were likely indirectly (i.e. multi-synaptically) driven by UOA photostimulation. Deep layer response onset (mean ± s.e.m: 30 ± 7 ms) lagged that in L4C, as would be expected if optogenetic activation first propagated through L4C before being synaptically relayed to deeper layers, via L4C-to-L5/6 connections. Averaged peri-stimulus time histograms (PSTHs) for the peri-pulse period on one example L4C and one L6 contact are shown in Fig. 4b. There was a significant pulse-by-pulse difference in onset latency across contacts (ANOVA, *p* < 10^−30^), as well as a significant pairwise difference across these two LEA recording sites (Tukey HSD test, *p* < 10^−6^; Fig. 4b, Right).

Figure 4c shows average peri-pulse PSTHs across all LEA contacts as a function of normalized cortical depth for exemplary whole array (top panels), single column (middle panels), and single μLED (bottom panels) stimulation at different intensities or stimulation site(s)-LEA separations. Increasing the total stimulus area at lower input intensities (panels in the left column of Fig. 4c) increased both the number of responsive contacts and the amplitude of driven responses, and shortened onset latencies. At higher input intensities (5 V, middle column), there was little change in these measures across large differences in total stimulated area. Decreasing the stimulus intensity for a fixed area (middle to left columns in Fig. 4c), or increasing the separation between the stimulated UOA site/s and the LEA for a fixed stimulus intensity (middle to right panels in the center and bottom rows of Fig. 4c) increased onset latencies across all contacts (mean latency ± s.e.m at 5 V and 3.2 V: 17 ± 1.7 ms and 25.4 ± 2 ms, respectively, whole array condition; 19.8 ± 1.4 ms and 37.5 ± 1.9 ms, C1 condition; 21.4 ± 2.3 ms and 74.1 ± 1.6 ms, C1-R8 condition; mean latency ± s.e.m at 5 V: 47.6 ± 4.3 ms and 59.4 ± 4.1 ms for C3 and C1-R6 conditions, respectively). Calculating onset latency on a pulse-by-pulse basis and looking at the effects on latency of cortical depth, stimulation pattern, and stimulation intensity, we observed significant main effects of pattern and intensity, as well as significant two-way and three-way interactions between all three factors (ANOVA, all *p* < 10^−4^). Limiting our analysis to each UOA stimulation pattern (that is, either whole array, single column, or single site), we observed a significant effect on latency of intensity and distance from the LEA for the single column conditions in Fig. 4c (ANOVA, all *p* < 10^−4^), and a significant effect of distance for the single µLED conditions (ANOVA, *p* = 0.03). Furthermore, in many conditions, pairwise comparisons across contacts revealed a significantly delayed response onset in deep layers relative to mid-layers for most conditions in Fig. 4c at an input intensity of 5 V, and for some conditions at 3.2 V (Tukey HSD, all *p* < 0.01; Supplementary Fig. 8). This time lag varied with intensity and separation between stimulation and recording sites, increasing at lower intensities and greater distances. There was also a significant difference in onset latency between mid- and superficial layers in some conditions (C1 at 5 V, whole array at 5 V and 3.2 V; Tukey HSD, all *p* < 0.01; Supplementary Fig. 8). Notably, however, when the whole µLED array was stimulated at the highest intensity (7.8 V), there was no significant difference in onset latencies between deep and middle layers, again suggesting the deep layer activation in this condition could have been caused by light spreading through deeper tissue (Supplementary Figs. 5d and 8).

**Fig. 5:**
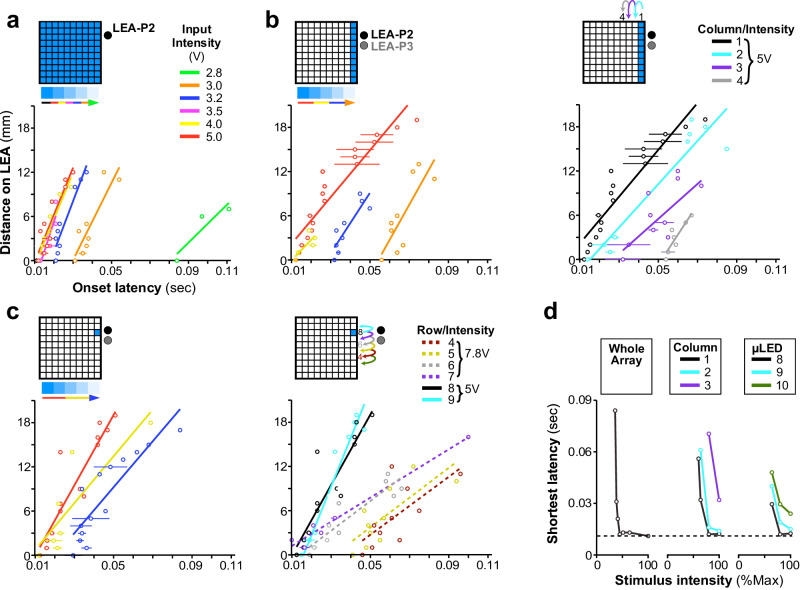
Population onset latencies as a function of UOA stimulation intensity and spatial pattern. **a** Distance on the LEA of each contact from the contact with the fastest onset latency is plotted against onset latency; lines are linear fits. Each line is from simultaneous stimulation throughout the whole µLED array at each indicated intensity (*n* = 125 – 185 pulses per condition). **b** Left: Effect of varying photostimulation intensity for a fixed column (C1) (*n* = 155–205 pulses per condition). Right: effect of varying stimulated column (C1–C4) for a fixed photostimulation intensity (5 V) (*n* = 155–235 pulses per condition). Either lowering intensity for a given column or increasing the distance between an activated column and the LEA had similar effects on the latency of network activation. **c** As in **b**, but for a single μLED stimulation condition. Left: photostimulation intensity was varied for a fixed µLED (C1-R8) (*n* = 160–175 pulses per condition); Right: the stimulated µLED was varied along column 1 (from row 3–9) at a fixed intensity (5 V for µLEDS in rows 8–10, but 7.8 V for those in rows 4–7, as lower intensities did not evoke a response from many of these latter µLEDs) (*n* = 160–255 pulses per condition). **d** The shortest onset latency across all intensities (here expressed as percent of max- see legend in Supplementary Fig. 5c for corresponding input voltage) is plotted for the whole array condition (Left), and selected columns (Middle) or μLEDs (Right). In all panels, error bars represent standard error of the mean.

To quantify the effects of UOA stimulation pattern and intensity on onset latencies across the population (*n* = 33 significantly responsive contacts, across 2 LEA penetrations), we first calculated the distance between each LEA contact and the contact with the shortest onset latency, then regressed this distance on onset latency, separately for each unique combination of UOA stimulation site(s) and intensity. This analysis revealed two main effects. (1) Onset latency decreased significantly across all contacts with increasing stimulation intensity (ANOVA, main effect of intensity, all *p* < 0.01; Figs. 5a, b, Left, c, Left) and proximity between the UOA stimulation site and LEA recording sites (ANOVA, main effect of row or column on UOA, all *p* < 10^−4^; right panels in Fig. 5b, c). (2) Onset latency increased significantly with contact distance, on the LEA, from the fastest contact (Fig. 5a–c, main effect of distance on the LEA, ANOVA all *p* < 0.01), suggesting that the more distant contacts were activated indirectly via inter-laminar networks. However, for stimulation of the whole UOA at higher intensity (7.8 V), evoked responses had similar onset latencies across the LEA (thus, across V1 layers; Supplementary Figs. 5e and 8 top right).

Across the three categories of UOA stimulation (whole array, column, and single μLED), only for the whole array and single µLED conditions did we observe a significant interaction between the effects of distance along the LEA and UOA photostimulation intensity on onset latency (Fig. 5a, c, Left; both *p* < 0.05, ANOVA). In these conditions, lowering photostimulation intensity decreased the slope of the LEA distance-onset latency curves, indicating that the difference in onset latency with distance on the LEA increased at lower intensity. Additionally, for the single μLED condition, we also observed a significant decrease in the slope of the curves when stimulating at increasing UOA-LEA separation, but only when we moved the single μLED stimulus to sites that were far enough from the LEA to necessitate stimulation at the very highest powers used to elicit any response (dashed lines in Fig. 5c, Right, µLED in rows 4–7; ANOVA, LEA distance × UOA row × intensity interaction, *p* < 10^−3^). For the single-column condition, there was no significant interaction between LEA distance and either photostimulation intensity or UOA-LEA separation (Fig. 5b; ANOVA, all *p* > 0.09). Importantly, across all three photostimulation patterns (whole array, single columns, and single µLEDs) there was remarkable similarity in the timing of the fastest responses (Fig. 5d). Both increasing stimulus area and stimulating at UOA sites closer to the recording locations reduced the light intensity necessary to evoke responses at this latency, but did not result in shorter latencies. This is further evidence that the evoked MUA nearby LEA contacts was relayed indirectly following optogenetic activation at UOA tips, and that the timing of this activation depended upon both the location and area of optogenetically activated inputs.

In summary, by varying photostimulation intensity and/or number of stimulated sites, the UOA allows activation of single or multiple layers, while by varying the spatial separation between the site of UOA stimulation and that of a recording probe, the UOA allows optogenetic investigations of local vs. long-range intra- and inter-laminar circuits.

### In vivo testing: c-Fos expression

**Fig. 6:**
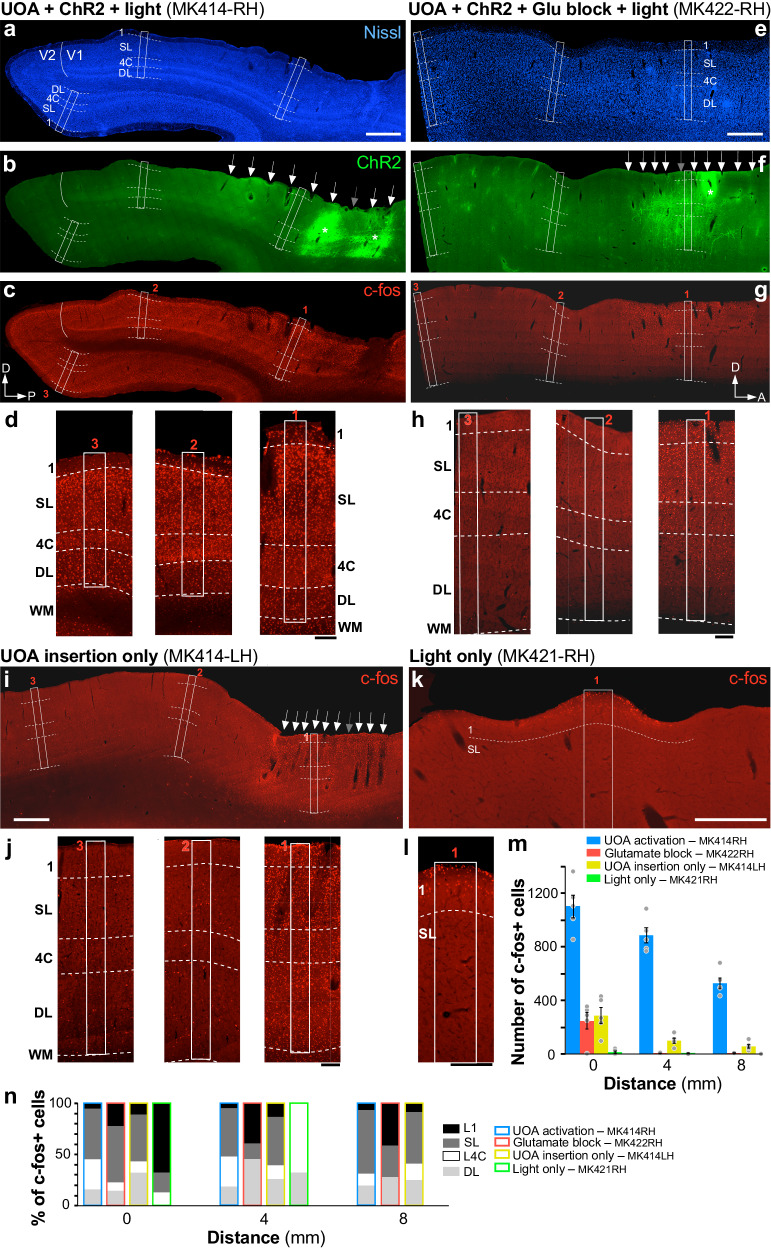
Local optogenetic activation through the uoa spreads through cortico-cortical networks. **a**–**c** Case MK414-RH (UOA activation). The same sagittal section encompassing parts of V1 and V2 is shown under 3 different fluorescent illuminations, to reveal Nissl stain (**a**), tdT/ChR2 expression (**b**; the red tdT fluorescence was converted to green for purpose of illustration), and c-fos IHC (**c**). White solid contour: V1/V2 border; dashed contours: layer boundaries (layers are indicated); white boxes: ROIs (numbered 1–3 in panel **c**) where c-fos+ cells were counted. White Arrows in (**b**) point to the visible damage caused by each UOA needle, while the gray arrow points to the likely location of one of the UOA needles that did not cause visible damage in this section. Asterisks in (**b**) mark the core of the viral injections, and sites of highest tdT/ChR2 expression. P posterior, D dorsal. C-fos expression in this case is observed throughout all layers (local) and across cortical areas (long-range). Scale bar in **a**: 1 mm (valid for **a**–**c**). **d** Higher magnification of c-fos IHC in and around each ROI. Scale bar: 0.2 mm. **e**–**h** Case MK422-RH (Glutamate block). Same as in (a-d) but for a different case in which an AMPA receptor antagonist was injected into the SMA prior to UOA insertion and photostimulation. The sagittal section is from the SMA. D dorsal, A anterior. Scale bars: 1 mm (**e**, valid for **e**–**g**); 0.2 mmm (**h**). Blocking AMPA receptors demonstrates that initial optogenetic activation is limited to the stimulated layers in the region of UOA insertion. **i**, **j** Case MK414-LH (UOA insertion-only). C-fos IHC in a sagittal section of SMA cortex (**i**) and at higher magnification in and around each ROI used for cell counts (**j**), in a case which only received UOA insertion in cortex not expressing ChR2, and no photostimulation. Scale bars: 1 mm (**i**), 0.2 mm (**j**). **k**, **l** Case MK421-RH (Light-only). Same as in **i**, **j**, but for a control case in which SMA cortex not expressing ChR2 only received surface photostimulation via an optical fiber-coupled laser and no UOA insertion. Here only one ROI is shown at higher magnification to reveal the few labeled cells in L1. Scale bars: 0.5 mm (**k**), 0.2 mm (**l**). The increases in cFos expression seen after full UOA activation of ChR2-expressing cortex cannot quantitatively be explained by device insertion or surface illumination. **m** Average number of c-fos+ cells across sections used for quantification, as a function of distance from the center of UOA insertion for the 4 different cases. Error bars: s.e.m. (**n**) Distribution of c-fos+ cells across layers at each distance.

To validate the performance of the UOA for large-scale photostimulation, we measured changes in c-fos expression, an immediate early gene whose expression rapidly increases when neurons are stressed or activated^40,41^. C-fos protein expression can be used as an indirect measure of the spatial pattern of neural activation. We analyzed patterns of c-fos expression using immunohistochemistry (IHC) (see “Methods” section) in two control and two experimental hemispheres from 3 animals. All four cases are shown in Fig. 6. Qualitatively, the cortical damage caused by the UOA insertion appeared comparable or even milder than that typically observed after explantation of a standard UEA^42–44^. Damage appeared limited to the cortical site of shank penetration (the holes in the tissue left by the shanks of the explanted array are marked by arrows in Fig. 6b, f, i), with no obvious differences in the quality of Nissl stain between the inter-shank regions and control distant tissue. However, quantitative microscopic analysis will be necessary for a detailed assessment of acute cortical damage.

In one experimental case (MK414-RH), a “passive” UOA (lacking an integrated µLED array) was implanted in a ChR2/tdT-expressing region of V1 (Fig. 6a, b). We stimulated the deep layers through a subset of needles, using a collimated, fiber-coupled, 473 nm laser, while shielding from light surrounding cortex and other portions of the UOA (see Methods). Histological analysis revealed that the UOA, in this case, was inserted at an angle (due to brain curvature), its needle tips ending at the bottom of the superficial layers, anteriorly, and in progressively deeper layers, posteriorly (most tips being in L4C, only the most posterior ones reaching L6) (Fig. 6a, b). C-fos positive (c-fos+) cells were found throughout V1 (Fig. 6a, c, d), as well as in V1 recipient extrastriate areas, including V2 (Fig. 6a, c, d), V3, and MT. This extensive pattern of elevated c-fos expression was likely induced by a combination of direct optogenetic activation and indirect subsequent synaptic activation.

To test the hypothesis that elevations in c-fos levels were due to both direct and indirect effects, we repeated the experiment in a different animal (MK422-RH) in which we greatly reduced glutamatergic neurotransmission via application of the AMPA receptor antagonist NBQX to ChR2-expressing cortex prior to passive-UOA insertion and photostimulation. Most of the UOA’s needle tips, in this case, only reached the bottom of the superficial layers (Fig. 6e, f). We also performed two additional experiments, to control for the potential of elevated c-fos expression being induced by either UOA insertion or stray photostimulation, respectively. In case MK414-LH, we inserted a passive UOA in the supplementary motor area (SMA) not expressing ChR2, and euthanized the animal 4 hours later without stimulating through the array. Histological analysis revealed that the UOA was fully inserted in this case (tips reaching L5; Fig. 6i). In case MK421-RH, instead, we only performed surface photostimulation of SMA cortex not expressing ChR2 and which received no UOA insertion (Fig. 6k).

To quantify c-fos expression across our various manipulations, we counted c-fos+ cells in 3 regions of interest (ROIs) encompassing all cortical layers. One ROI was centered in the region of UOA insertion and/or light stimulation; the other two ROIs were located 4 and 8 mm, respectively, from the first (white boxes numbered 1–3 in Fig. 6a–l; see “Methods” section). Figure 6m plots the average number of c-fos+ cells across samples, as a function of distance from the UOA insertion site, while Fig. 6n shows the laminar distributions of c-fos+ neurons at each distance. We found significant local (involving all layers) and long-range c-fos expression only when photostimulation of ChR2-expressing cortex was performed via the UOA (MK414-RH; Fig. 6c,d,m,n). Blocking glutamate neurotransmission prior to photostimulation prevented long-range c-fos expression, and reduced its expression by 5 fold in the area of UOA stimulation, where it was largely confined to the directly stimulated layers (mostly superficial) near the UOA tips (MK422-RH; Fig. 6g, h, m, n). Simply inserting the UOA (without any subsequent photostimulation) led to as much local c-fos expression as in the glutamate block case, but with greater inter-laminar (involving all layers), as well as intra- and inter-areal long-range spread (MK414-LH; Fig. 6j, m, n). This suggests that neurons activated by the insertion trauma also indirectly activated downstream networks. Finally, surface photostimulation of cortex not-expressing ChR2, without UOA insertion, caused virtually no c-fos expression, except for a few cells in L1 and upper L2 (MK421-RH; Fig. 6l–n). Statistical analysis (one-way ANOVA with Bonferroni corrected post hoc comparisons) revealed a significant difference in the number of c-fos+ cells in each ROI between the full experimental case (MK414-RH) and all others (all *p* < 0.001 at all distances for all pairwise comparisons). There was no significant difference in the number of c-fos+ cells between the glutamate-block and UOA-insertion-only cases at any distance (*p* > 0.23 at all distances), and both these cases differed significantly from the light-only case at 0 mm distance (*p* < 0.05 for all comparisons). Finally, the number of c-fos+ cells decreased significantly with distance for cases MK414-RH (*p* < 0.001), MK422-RH (*p* = 0.001), and MK414-LH (*p* = 0.003), but not for the surface stimulation control (case MK421-RH) (*p* = 0.079).

## Discussion

We have developed and validated a novel device, the UOA, which has the potential to further optogenetic research in NHPs. Current optogenetic approaches in NHPs permit light delivery either over a large superficial area^9,23^, or to deeper tissue over a small area^25–27,38^. Multi-site probes for larger volume stimulation have also been developed, and combined with single^45^ or multisite^46,47^ electrical recordings, but these approaches are typically cumbersome to assemble and don’t easily scale to precisely target multiple small tissue volumes. The UOA combines the advantages of all these approaches and retains millisecond-scale temporal resolution. It allows for both focal and larger-scale neuronal activation of single or multiple deep layers, simply by varying the number of simultaneously activated µLEDs and/or the light irradiance. Moreover, although here we only used the needle-aligned µLED array for deeper layer activation, the integrated interleaved interstitial µLED array will allow for selective photostimulation of superficial layers, either independently or in conjunction with deep layers.

By design, the UOA is intended to achieve sufficient spatial resolution for cortical application in NHPs, and eventually humans, and is, thus, ideal for addressing neuroscience questions that require large-scale manipulations of deep and/or superficial cortical layers. Here we have demonstrated that the UOA, used as a stimulation-only device in conjunction with LEA recordings, can be used to study inter-laminar interactions. We were able to localize photostimulation to single or multiple cortical layers by varying light intensity. Similarly, varying insertion depth (or shank length) enables targeted selection of layers. Relative differences in onset latency of evoked responses could be used to distinguish distinct network activity patterns following different patterns of UOA stimulation. For example, at low light irradiance, direct neuronal activation was initially localized to layers nearest optrode tip termination before spreading trans-synaptically to other layers. Increasing light irradiance reduced or eliminated these latency differences. Similarly, firing rates in L4C showed a non-monotonic dependence on stimulation intensity, suggesting response amplitude could be used to identify local activation of higher threshold inhibitory networks.

We should note that we were unable to evoke optogenetic responses in one LEA penetration. Post-mortem histology revealed that the UOA sites nearest this recording location were in a region with minimal tdT/ChR2 expression and the LEA was inserted farther from the UOA compared to the other two LEA penetrations (Supplementary Fig. 4a). In general, when recording with an electrode or electrode array placed adjacent to the UOA, failure to evoke responses could be due to some combination of factors related to: opsin expression, UOA-electrode separation, the pattern of direct and multi-synaptic connections between neurons at the UOA and recording location(s), and optical power across UOA sites (nearby and distant to the recording site(s)). Thus, experiments with the UOA should ensure a sufficiently large volume of opsin expression, good alignment of UOA placement within the transfected region, and multiple electrode penetrations to yield reliable effects.

By varying the distance between the stimulation site/s on the UOA and the recording electrode, local versus long-distance intra-areal interactions can be studied. Moreover, used in conjunction with c-fos IHC, we were able to identify multi-synaptic interactions within and beyond the photostimulation area. Photostimulation via the UOA increased c-fos expression over distances much > 8 mm (well beyond the stimulated cortical area), but spiking activity could not be evoked beyond ~3 mm from the stimulated site, indicating c-fos expression revealed subthreshold activity induced by network interactions. This is consistent with previous demonstrations of c-fos expression several synapses away from an electrically stimulated site. Thus the UOA in conjunction with c-fos IHC can be used for functional mapping of neuronal circuits^40^.

In all optogenetics studies, it is important to control for possible thermal confounds, such as a local increase in brain temperature due to the µLEDs heating up when activated. This concern arises with implantable devices^48^ both because of temperature-induced tissue damage^49^ and changes in spiking activity^24,50^. It is generally assumed that tissue damage is negligible for temperature increases < 1°C^29,51^. One difference of the UOA compared to other implantable μLED devices is that the heat-generating μLEDs are mounted on the topside of the device and external to tissue, compensating for the fact that the low optical coupling efficiency requires higher drive currents than for optogenetic devices based upon embedded μLEDs on implantable shanks^48^. Detailed thermal simulations showed that the intervening thermally-insulating layers of dura-gel and brain tissue (combined thickness ~1.5 mm) caused *a* ~ 1 s delay in the temperature ramp at the stimulation site in L4, so that the bulk of the temperature rise (and subsequent fall) occurred during the inter-trial interval and not during the trial period. These simulations also showed that peak temperature rise could be held below 1 °C, due in part to heat spreading by the interposer layer. Additional analysis of spiking rates during the inter-trial interval showed some modulation from background activity, which could be temperature mediated, but only when the whole array was activated at the highest intensity; and even for this condition, spiking activity had returned to baseline by the end of the inter-trial interval (i.e., prior to the next trial). Furthermore, tissue heating can also occur from direct light absorption^24^. The maximum irradiance used here was under 4 mW/mm^2^, which would imply a maximum local increase in temperature of ~0.03 °C, based upon a prior report^24^. These results strongly suggest our results were not affected by thermal increases.

Future applications, beyond those demonstrated here, could involve functional investigations of inter-areal circuits, when UOA stimulation in one cortical area is coupled with recordings in a different area. Importantly, despite its limited shank length (~2.5 mm maximum), the UOA can also be employed to study cortico-subcortical interactions, e.g., through modulation of axon terminals of deep nuclei within cortex, and recordings of postsynaptic cortical neurons in the same cortical area and/or layer.

At present, the UOA is an optical stimulation-only device. Future generations of the device are planned that include multi-color photostimulation and recording sites at the optrode tips. The technical challenge to integrating neural recording directly onto the device lies in establishing an electrical path between a 10×10 array of bond pads on the backside of the device and the tips of the shanks where recording would be performed. An electrical path that traverses the µLED substrate, interposer, and the entire shank length needs to be established, requiring some reworking of the device structure, such as utilizing conducting vias through the silicon interposer. A further challenge is to minimize potential stimulus artifact, which has two components – capacitive coupling between µLED drive lines and recording lines, and photoelectric effects. Capacitive coupling can be mitigated through optimized layout and state-of-the-art shielding approaches^33^. Photoelectric effects can be mitigated by using transparent conductors and low-intensity, below-bandgap, illumination to avoid interband transitions^52^. With the UOA, the use of a transparent conductor is needed at least at the tips, through which light is transmitted. There has been significant work in the field on neural electrodes for recording and stimulation based on transparent conductors, such as ITO films^53–56^, doped PEDOT coatings^54,57,58^ and films^59^, graphene^60–62^, and metallic meshes^63^. These materials have been used in vivo successfully in acute and semi-chronic application in µECoG^55,56,58,61,62,64^ and neural probe^54,57,65^ devices. However, it is not clear that any material studied to date meets the dual requirements of high transparency and long-term chronic in vivo stability. These issues do affect the broader community, and continued efforts should be devoted to optimization and new materials systems. In conclusion, the UOA, as currently conceived, will enable studies addressing fundamental questions in neuroscience, e.g., the role of corticocortical feedback and cortical layers in the model systems closest to humans. One obvious first use of the device in NHP behavioral experiments could be to modulate selectively the deep-layer cortico-cortical feedback connections that are believed to play a role in selective attention^16^, visual context^15^, and predictive coding^66,67^. As a test of its therapeutic potential, the resolution of the phosphene map generated via optogenetic stimulation of V1 neurons through the UOA could be compared with that generated through conventional microsimulation. As many human neurological and psychiatric disorders have been linked to abnormalities in cortical circuits^4,5^, this technology can improve our understanding of the circuit-level basis of human brain disorders, and will pave the way for a new generation of precise neurological and psychiatric therapeutic interventions via cell-type-specific optical neural control prosthetics.

## Methods

### Device fabrication, characterization, and benchmarking

Fabrication and testing of the first generation UOA devices was previously reported^28,68^. The second-generation devices used in this study included an optical interposer layer that limits emission from the µLED array to the shank sites for illumination of deep cortical tissue.

### Fabrication

A 2-mm-thick, 100 mm diameter Schott Borofloat 33 glass wafer used to construct the optrode needles was anodically bonded to a freshly cleaned 0.1 mm thick, 100 mm diameter intrinsic Si wafer serving as an optical interposer. The Si and Borofloat wafers were coarsely aligned, and bonding performed using an EVG 520 anodic bonder. The optical vias were patterned in the Si interposer by deep reactive ion etching (DRIE) using a Bosch process. A 10-µm-thick AZ9260 soft mask was photolithographically patterned to define the array of 80 × 80 µm^2^ optical vias for shank and interstitial illumination for the DRIE process. The bonded wafer was then sub-diced into *modules* of 9 to 16 UOAs using a DISCO 3220 dicing saw.

UOA modules were mounted to a carrier wafer using WaferGrip™ (Dynatex International, Santa Rosa, CA). The glass shanks were cut with the DISCO 3220 using the previously reported process^28,68^. Briefly, beveled blades were first used to generate pyramidal tips on the surface, followed by standard profile blades to form the shanks. The shanks on a module were then etched to a nominal 110 µm thickness using a mixture of hydrofluoric (49%) and hydrochloric (37%) acid in a 9:1 ratio. The die was then demounted and cleaned, and the shanks were smoothed to decrease light scattering using a 725 °C heat treatment for 2 h in a vacuum furnace. UOA modules were then singulated into individual 4 × 4 mm^2^ UOAs using the DISCO 3220.

Arrays of µLEDs on thinned (150 µm) sapphire substrates, from the Institute of Photonics at University of Strathclyde, were integrated with the UOA using closed-loop optical alignment to the optical vias on individual UOAs at Fraunhofer IZM (Berlin, Germany)^28^, and bonded using index-matched epoxy. At the University of Utah, passive matrix µLED pads (10 common anode and 10 common cathode) were wire bonded to an ICS-96 connector (Blackrock Microsystems, Salt Lake City, UT) using insulated gold alloy wire. The wire bundle and backside of the UOA were then potted in NuSil MED-4211 silicone, respectively, followed by overcoating with a 6µm-layer of Parylene C. The passive matrix approach reduces the number of connections, at the cost of limiting the patterns that can be displayed via simultaneously addressing multiple µLEDs. For example, illumination patterns consisting of an individual µLED, one or more horizontal lines, one or more vertical lines, and individual rectangles/squares can be produced. Addressing diagonal patterns or simultaneous horizontal and vertical lines will result in filled rectangular illumination.

### Bench testing

To characterize the electrical and optical performance of the finalized devices, the latter were attached to a custom switchboard for matrix addressing the individual optrode shanks. The switchboard consisted of a matrix arrangement of parallel-connected mechanical switches and electrical relays, 10 sets for the anodes and 10 sets for the cathodes. This enabled both manual and automated activation of individual optrode shanks or optrode patterns. For the automated activation and testing, the relays were connected to Arduino boards that received commands from the lab computer. To prevent voltage spikes originating from the switching of the channels from damaging the µLEDs, the anode paths also contained a small filter circuit consisting of capacitors and Zehner diodes (break-down voltage: 8.2 V). For the automated testing, the UOAs were inserted into the opening of an integrating sphere that was, in turn, connected to a photodetector and power meter (Newport 2832-C Dual-Channel Power Meter). The calibration factor of the integrating sphere was determined using a fiber-coupled LED prior to the experiment. Then the UOAs were connected to the switchboard, and the latter was connected to a source measure unit (Keithley 236 Source Measure Unit) for the measurement. The automated characterization was conducted as follows: the switchboard’s Arduino boards received the command to switch to an individual optrode shank using the relays. Then the source measure unit applied a voltage pulse measurement pattern (pulse length 100 ms, pause between pulses 1900ms to prevent heat buildup) sweeping the voltage from 0 to 7.2 V (or until the compliance current of 100 mA was reached) with each pulse increasing by 100 mV. For each pulse, the resulting current and the output optical power were recorded; the optical power was then corrected using the integrating sphere calibration factor. This was repeated for each individual optrode shank of the device for a full characterization.

To ensure the stability of the device for an acute in vivo experiment, additional voltage transient measurements were made before and after a 48-hour soak test in phosphate-buffered saline (PBS) at 37 °C. Further, an electrode was immersed in solution to verify encapsulation integrity, as evidenced by lack of shorting to solution.

### Modeling

To understand light spread in tissue, the optical output of the device was modeled using commercial ray-tracing software (Optics Studio 12, with which light rays are traced from the µLED source through the interposer and needle and into tissue using non-sequential mode). This model has been described previously^28^. Brain tissue was modeled using a Henyey-Greenstein scattering model, with a scattering coefficient of 10 mm^−1^, absorption coefficient of 0.07 mm^−1^, and anisotropy of 0.88^51^. Each needle was modeled individually using its measured optical output at the given voltage level. To generate the cross-section images from a simultaneously illuminated column (Fig. 1g), the light output from the 10 needles in that column were summed.

A finite element analysis (FEA) software package (Comsol Multiphysics) was used to model the thermal performance of our device (material properties are included in the Table presented in Supplementary Fig. 6. A 3D model of the device geometry was created with the µLED acting as a heat source. It was assumed that 90% of the electrical power is converted to heat in the µLED structure. A brain perfusion rate of 50 mL per 100 g of tissue per minute was also included. Various µLED drive currents, pulse widths, and frequencies were tested. Multiple µLEDs were also illuminated simultaneously. The thermal model was consistent with measures of the external temperature of the device using a thermal camera during device operation in vivo.

### Animals

A total of 3 adult female Cynomolgus monkeys (*Macaca fascicularis*) were used in this study. The left hemisphere of one animal (case MK421-LH) was used for the in vivo electrophysiological testing of the active UOA (integrated with the µLED array). The right hemisphere from the same animal (MK42-RH), and 3 hemispheres from 2 additional animals (MK414RH and LH, and MK422-RH) were used for c-fos testing of the passive UOA (i.e., without an integrated µLED array). All procedures complied with the ethical regulations set by the National Institutes of Health Guide for the Care and Use of Laboratory Animals and were approved by the University of Utah Institutional Animal Care and Use Committee.

### Survival surgical procedures and viral injections

Animals were pre-anesthetized with ketamine (10 mg/kg, i.m.), intubated, placed in a stereotaxic apparatus, and artificially ventilated. Anesthesia was maintained with isoflurane (1–2.5% in 100% oxygen). Heart rate, end tidal CO_2_, oxygen saturation, electrocardiogram, and body temperature were monitored continuously. I.V. fluids were delivered at a rate of 3–5/cc/kg/hr. The scalp was incised and a craniotomy and durotomy were performed over area V1 (*n* = 2 animals, MK421-LH and MK414-RH), or rostral to the precentral gyrus, roughly above the supplementary motor area (SMA; *n* = 1, MK422-RH).

We injected a 1:1 viral mixture of AAV9.CamKII.4.Cre.SV40 and AAV9.CAG.Flex.ChR2.tdTomato (Addgene Catalog #s: 105558, and 18917, respectively). We have previously found that this method nearly eliminates retrograde expression of transgenes^9^. This allowed us to restrict expression of ChR2 to excitatory neurons within V1 in order to simplify interpretation of the circuitry responsible for any optogenetic effects. The viral mixture was slowly (~15 nl/min) pressure-injected (250-350 nl repeated at 2 or 3 cortical depths between 0.5 and 1.5 mm from the cortical surface) using a picospritzer (World Precision Instruments, FL, USA) and glass micropipettes (35-45 µm tip diameter). After each injection, the pipette was left in place for 5–10 min before retracting, to avoid backflow of solution. A total of 5-6 such injections, each 500-750 nl in total volume, and spaced 1.5–2 mm apart, were made in two animals (MK421-LH, MK414-RH) while the third animal (MK422-RH) received 2 ×1,050 nl injections. These injections resulted in a region of high viral expression roughly 4–6 mm in diameter (as an example see Supplementary Fig. 4a, Right). Following viral injections, a sterile silicone artificial dura was placed on the cortex, the native dura was sutured and glued onto the artificial dura, covered with Gelfoam to fill the craniotomy, and the latter was sealed with sterile parafilm and dental acrylic. Anesthesia was discontinued and the animal returned to its home cage. After a survival period of 5–10 weeks, to allow for robust ChR2 expression, the animals were prepared for a terminal UOA photostimulation procedure.

### Terminal surgical procedures and UOA insertion

Monkeys were pre-anesthetized and prepared for experiments as described above. Anesthesia and paralysis were maintained by continuous infusion of sufentanil citrate (5–10 µg/kg/h) and vecuronium bromide (0.3 mg/kg/h), respectively. These drugs are the drugs of choice for these experiments, due to their limited impact on neuronal response properties^69^ and ease of use, respectively. Vital signs were continuously monitored for the duration of the experiment, as described above. Following suture removal and scalp incision, the craniotomy and durotomy were enlarged to allow space for device implantation, and ChR2 expression was verified in vivo using a custom fluorescent surgical microscope (Carl Zeiss, GmbH; Fig. 2b).UOAs were positioned over cortical regions exhibiting high tDT/ChR2 expression (again, as determined with the use of a surgical fluorescence microscope – e.g., Figs. 2b and 6b, f) and then inserted using a high-speed pneumatic hammer designed to minimize tissue damage during insertion of the Utah Electrode Array^70^ (Electrode Inserter System - Blackrock Neurotech, Salt Lake City, UT). We found several parameters of the method critically affected both UOA insertion accuracy and tissue response. First, we found that insertion pressure and pulse width affected the depth of insertion – for all experiments reported here, we used 20 psi with a pulse width of 30 ms. Note that the exact values that produce maximal insertion to the desired depth could differ across inserter systems or uses. The pressure and pulse settings should first be properly calibrated by testing (e.g., against a gloved finger) that device engagement results in a single strike of the insertion hammer. Second, when using the pneumatic inserter, we found that first placing a drop of sterile saline on a thin periosteal elevator and then gently placing the elevator against the backplane of the UOA and striking the elevator with the inserter hammer ensured a clean delivery of the UOA into cortex with no pullback of the UOA during retraction of the hammer (which could be caused by surface tension at the hammer/UOA backplane interface). Third, we found that spacer length affected the mean insertion depth; in all experiments reported here, we used a 1 mm spacer. For future use, experimenters will need to select a combination of UOA shank length and spacer length that suits the demands of the targeted tissue (determined in advance through either preoperative structural imaging or published reports of cortical thickness in the area of interest). Finally, to minimize potential tissue damage from excessive pressure of the UOA backplane, we aimed for an initial partial insertion of the UOA. In two of the cases used for the c-fos imaging experiments, after partial insertion, the UOA was fully inserted by applying gentle pressure to the backplane (e.g., with forceps or a periosteal elevator).

### Photostimulation

We implanted two types of UOA devices: (i) a 10×10 UOA with fully integrated μLED arrays (also referred to as “active” device; *n* = 1 device in 1 animal, MK421-LH; see Fig. 2a–c), and (ii): 10 × 10 UOAs with an optical interposer integrated into the sapphire backplane, but with no μLED array for light delivery (referred to as “passive” devices; *n* = 3 devices in 3 hemispheres from 2 animals, MK414-RH, MK414-LH, MK422-RH). The active device was used for electrophysiological testing experiments, while the passive devices were used for the c-fos experiments.

### Active device (electrophysiology)

Photostimulation with the active UOA occurred via the integrated µLED array. Photostimulation parameters were 5 Hz, 100 msec-pulse duration for 1 sec, followed by 1.5-21 sec inter-trial interval (longer intervals were used at the higher photostimulation intensities). We varied the spatial pattern (single µLED along column 1, entire single columns, and all µLEDs across the entire UOA) and intensity (from 2.8 to 7.8 V input intensity) of photostimulation as described in the Results section.

For the in vivo experiments, the switchboard was upgraded two-fold: first, transistors were added to the cathode channels to allow for turning the device on and off based on an external TTL trigger, delivered via the Cerebus recording system (Blackrock Neurotech, Salt Lake City, UT). However, we found that turning on the optrodes using the trigger signal directly induced too strong a capacitively-coupled voltage signal in the recordings. Therefore, as a second upgrade, an additional Arduino board with digital-analog-converter was added that received the external trigger and introduced rise and fall times to the square wave. This reduced the capacitively-coupled interference to a level below measurable when both the LEA and the UOA were in close proximity in 1xPBS solution prior to the in vivo experiments. During an experiment, the voltage for the UOA was supplied by a lab power supply via the switchboard; switches were operated manually to define the required patterns ((a detailed schematic and description of the switchboard and matrix addressed μLED array is provided in Supplementary Fig. 9).

### Passive devices (c-fos)

Selective photostimulation via passive devices was obtained by illuminating a subset of UOA needles with an appropriately positioned fiber-coupled 473 nm laser (400 µm multimode optic fiber, ThorLabs Newton, NJ; laser: Laserwave, Beijing, China) held in place with a stereotaxic tower. We used a collimating lens (ThorLabs, Newton, NJ) to restrict spot size to ~1.5 mm in diameter. To shield stray light, we covered any exposed tissue around the illuminated area, as well as the non-illuminated portions of the UOA, with an opaque (black) artificial dura. For each UOA we stimulated 2 or 3 separate sites. At each site we used phasic photostimulation (50 Hz for 2.5 min, 2.5 min pause, and 20 Hz for an additional 2.5 min; pulse duration was 10 ms) at 3.8 mW power output (corresponding to an estimated irradiance of 15-19 mW/mm^2^).

### Electrophysiological recordings

Extracellular recordings were made in V1 with 24-channel linear electrode arrays (LEAs; V-Probe, Plexon, Dallas, TX; 100μm contact spacing, 300μm from tip to first contact, 20μm contact diameter). The LEAs were inserted into the cortex next to the UOA to a depth of 2.4–2.6 mm, slightly angled laterally (towards the UOA) and posteriorly. We made a total of 3 penetrations (LEA-P1-P3; Supplementary Fig. 4a), of which only LEA-P2 and LEA-P3 provided useful data. After UOA and LEA were inserted into the cortex, we applied a layer of Dura-Gel (CambridgeNeuroTech, Cambridge, UK) over the cortex and UOA, to prevent the cortex from drying and stabilize the recordings. A 128-channel recording system (Cerebus, Blackrock Microsystems, Salt Lake City, UT) was used for standard signal amplification and filtering. Multi-unit spiking activity was defined as any signal deflection that exceeded a voltage threshold (set at 4 x the SD of the signal on each channel). Threshold crossings were timestamped with sub-millisecond accuracy. We did not record responses to visual stimuli but only to UOA photostimulation performed as described above; thus, the monkey’s eyes were closed during the duration of the experiment.

### Analysis of electrophysiological data, statistics and reproducibility

We analyzed MUA responses from a total of 45 contacts deemed to lie within the parafoveal representation of V1 in two penetrations (out of 3 total) for which neural activity was modulated by photostimulation via the active UOA. For the results presented in Figs. 3–5, quantitative analysis was limited to contacts on which MUA was stimulus modulated (one-way ANOVA comparing spike rates during full one-second photostimulation trials with spike rates during control periods of equivalent duration, *p* < 0.01).

To quantify the change in MUA firing rates, relative to background, during photostimulation we calculated firing rates for all pulse epochs within all trials and then compared them to the average background rate. To estimate the preference at each recording site for stimulation across the full range of tested UOA locations (Fig. 3), we regressed average evoked-responses on UOA stimulation site and intensity. Preliminary analyses had revealed a non-monotonic relationship between stimulation intensity and response on many contacts (cf. Fig. 2f), thus we included a quadratic term in the regression model.

### CSD analysis

For the CSD analysis shown in Fig. 2d-e, current source density (CSD) was calculated from the band-pass filtered (1-100 Hz) and pulse-aligned and averaged LFP in response to the first pulse in a train (to avoid adaptation effects), using the kernel CSD toolbox (kCSD Matlab)^71^. CSD was calculated as the second spatial derivative of the LFP signal, reflecting the net local transmembrane currents generating the LFP. The depth profile of the CSD was estimated by interpolating every 10μm. To facilitate comparisons across conditions, CSDs from different conditions were normalized to the standard deviation (SD) of the baseline (50 ms prior to first pulse onset) after subtraction of the baseline mean.

### Onset latency

To quantify the onset latency of MUA responses, we either: (i) calculated the average peri-stimulus time histogram (PSTH) from all pulse-aligned responses (e.g., Fig. 4) or (ii) estimated a PSTH separately for the response to each pulse (e.g., Supplementary Fig. 8). PSTHs were estimated via an adaptive algorithm in which the MUA raster was first convolved with a Gaussian kernel of fixed width (3 ms bandwidth). Kernel width was then adapted so that the number of spikes falling under the kernel was the same on average across the response (http://chronux.org^72^). We then subtracted the mean baseline response from the stimulus-evoked response. For each response measure, i.e., either the average or pulse-by-pulse PSTHs, we took the time at which the response reached 25% of the peak as the onset latency (results were qualitatively similar using 15% and 35% criteria). We report the time for the average PSTH to reach this criterion as the mean onset latency in Figs. 4–5. We used the time to criterion for the pulse-by-pulse PSTHs to test for differences in onset latency across contacts within and across UOA stimulation parameters (Figs. 4–5 and Supplementary Fig. 8).

### Statistical analysis

Stimulus-evoked firing rates were calculated from pulse-aligned or trial-aligned responses and baseline corrected (mean baseline activity subtracted). We determined responsiveness to stimulation via a one-way ANOVA comparing firing rates during the full 1-second trial period with inter-leaved control periods of equivalent duration; MUA at an LEA recording site was deemed responsive if there was a significant difference between stimulation and control trials at the *p* = 0.01 level. To estimate the selectivity of MUA for stimulation at different UOA sites we fit multiple linear regression models (with and without a quadratic term), with UOA column, row, and intensity as, categorical independent variables and pulse-aligned, baseline corrected, firing rates as the dependent measure. Rather than assessing differences in goodness-of-fit of the linear and quadratic models on a contact-by-contact basis, to test for differences across the population in the goodness-of-fit of models with- and without a quadratic term, we used a two-sample Kolmogorov-Smirnov test. We assessed the effects of varying UOA stimulation site and intensity on MUA response amplitude or onset latency using ANOVA models. In cases in which we observed a significant main effect in the ANOVA, to test for differences in the mean response measure across pairs of LEA contacts, for all possible pairings, we used the Tukey-Kramer test for multiple comparisons.

### c-fos experiments

We used 4 hemispheres from 3 animals for these experiments (MK414-RH and LH, MK422-RH, and MK421-RH). Two of these animals (MK422 and MK414) were prepared for a terminal experiment (as described above) 5 or 10 weeks, respectively, after the viral injections, and a passive UOA was inserted in regions of high tdT/ChR2 expression in the injected hemisphere. In one of these animals (MK422-RH), UOA insertion was preceded by glutamate block (see below). After UOA insertion, photostimulation was performed via an optical fiber-coupled laser through the UOA, as described above. Two additional hemispheres in 2 animals (MK414-LH and MK421-RH) were used as controls. Specifically, case MK414-LH received insertion of a passive UOA in non-opsin expressing SMA cortex, and was euthanized 4 hours following UOA insertion without receiving any photostimulation. As a separate control, in case MK421-RH we performed surface photostimulation of SMA cortex not expressing opsins, using a fiber-coupled laser and a collimating lens and the same photostimulation protocol described above for other c-fos experiments; no UOA was inserted in this case. In all animals, UOA insertion and/or photostimulation were performed after a 10-14-hour period of eye closure and at least 5 hours after completion of surgery, and the animals were euthanized 75 minutes after completion of the photostimulation protocol.

### Pharmacological blockade of local glutamate signaling

To compare changes in c-fos expression due to direct local optogenetic activation with indirect local and long-range changes due to synaptic increases in excitatory glutamatergic neurotransmission downstream of the directly activated neurons, in one case (MK422-RH) we applied the selective glutamate AMPA receptor antagonist 2,3-dihydroxy-6-nitro-7-sulfamoyl-benzoquinoxaline-2,3-dione (NBQX, 5 mM) (Tocris BioSciences). NBQX was applied topically prior to UOA insertion, by soaking a piece of Gelfoam placed over ChR2-expressing SMA cortex with 1 ml of the drug solution. The drug was allowed to passively diffuse through the cortical layers for 90 minutes, during which 100-200 µl of the solution were applied every 15 minutes to ensure saturation of the Gelfoam, after which the Gelfoam was removed and the passive UOA inserted over the region of glutamate block. Photostimulation was performed as described above for the passive device.

### Histology

On completion of the experiments, the animals were euthanized by an overdose of Beuthanasia (0.22 ml/kg, i.v.) and perfused transcardially with saline for 2–3 min, followed by 4% paraformaldehyde (PFA) in 0.1 M phosphate buffer for 20 min to fix the brain. The brains were post-fixed overnight in the same fixative, sunk in cryoprotectant 30% sucrose solution, and sectioned at 40 µm on a freezing microtome. The hemisphere used for electrophysiological testing of the active UOA (MK421-LH) was sectioned tangentially. One in 3 sections were wet-mounted and imaged for fluorescent tdT-label at 10x magnification. The same sections were then reacted for cytochrome oxidase (CO) to reveal cortical layers and the location of UOA and LEA insertions and shank or contact locations visible as discolorations in CO staining (Supplementary Fig. 4a Left).

All other hemispheres used for c-fos experiments were sectioned sagittally. One full series of sections (1:3) were immunoreacted for c-fos by overnight incubation in primary antibody (1:500 rabbit anti-c-fos, Ab 19089, Abcam, MA, USA) at room temperature, followed by 2 h incubation in near-infrared secondary antibody (1:200 donkey anti-rabbit IgG-AF647, Jackson ImmunoResearch, PA, USA) at room temperature. Sections were then wet-mounted, counterstained with blue fluorescent Nissl (1:100 N21479, Thermo Fisher Scientific, MA, USA) by dripping the solution onto the slide-mounted sections every 5 min for 20 min, rinsed, and coverslipped and sealed with CoverGrip™ Coverslip Sealant (Biodium, CA, USA).

### Tissue imaging

Imaging of tissue sections was performed on a Zeiss Axio Imager.Z2 fluorescent microscope (Zeiss, Germany) with a Zeiss X-cite 120 LED Boost light source, using a 10x objective and an Axiocam 506 mono camera (Zeiss, Germany). Image files were created and analyzed using Zen 2.6 Blue Software (Zeiss, Germany). The light intensity was set to 100%, and the exposure time for each channel was kept the same between images. The tangentially-sectioned hemisphere (MK421-LH) was imaged as described above. In all other cases, each sagittal section was imaged in 3 channels simultaneously, one channel for tdT/ChR2 (red- but note the color was artificially changed to green in Fig. 6b,f), one channel for Alexa-647-c-Fos (far-red), and the third channel for 435-455 Nissl (blue).

### Analysis of c-fos expression

To quantify c-fos expression, c-fos+ cells were plotted and counted in sampled areas, using Neurolucida software 2006 (Microbrightfield Bioscience, VT, USA). For each case, we selected for counts 5 sections spaced 1 mm apart encompassing the area of UOA insertion and/or photostimulation (for the light-only case). In each section, we plotted and counted cells within three 200µm-wide windows spanning all cortical layers, one positioned at or near the center of the UOA insertion region (or of phtostimulation-only), and the other two located at distances of 4 mm and 8 mm, respectively, from the center of the UO insertion (Fig. 6). Thus, a total of 15 regions of interest (ROIs) were counted for each case. The laminar distribution of c-fos+ cells was analyzed by tracing the layers on the Nissl stain and counting the number of c-fos+ cells within each layer in Neurolucida. Statistical differences in c-fos+ cell counts among experimental and control cases, and across distances were estimated using a one-way ANOVA with Bonferroni corrected post hoc comparisons.

### Reporting summary

Further information on research design is available in the Nature Portfolio Reporting Summary linked to this article.

### Supplementary information

Peer Review File

Supplementary Information

Description of Additional Supplementary Files

Supplementary Data

Reporting Summary

## Data Availability

The data presented here will be provided upon reasonable request to the corresponding authors. Source data for the figures are provided with the paper. The source data for the graphs in the figures and Supplementary Figs. can be found in the Supplementary Data file.

## Code availability

All custom software used for modeling will be provided upon reasonable request to the corresponding authors. All analysis code can be found at: https://github.com/visCortexLabUtah/utahoptrodearray-neurophysAnalysis.
