# Supplementary material for: An optrode array for spatiotemporally-precise large-scale optogenetic stimulation of deep cortical layers in non-human primates
Source: Commun Biol. 2024 Mar 14;7:329. doi: 10.1038/s42003-024-05984-2 (PMC10940688; doi:10.1038/s42003-024-05984-2)
Supplement: Supplementary file 2 — Supplementary Information [file 42003_2024_5984_MOESM2_ESM.pdf]

SUPPLEMENTARY INFORMATION FOR:

An Optrode Array for Spatiotemporally Precise Large-Scale Optogenetic Stimulation of Deep Cortical Layers in Non-human Primates

Andrew M. Clark, Alexander Ingold, Christopher F. Reiche, Donald Cundy III, Justin L. Balsor, Frederick Federer, Niall McAlinden, Yunzhou Cheng, John D. Rolston, Loren Rieth, Martin D. Dawson, Keith Mathieson, Steve Blair, and Alessandra Angelucci

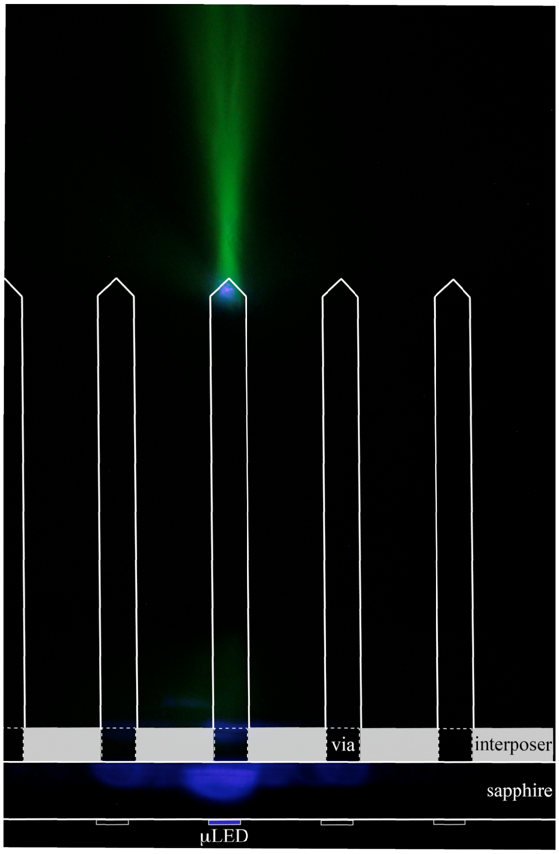

Supplementary Figure 1

Absence of optical crosstalk due to interposer
A UOA device with needle shanks immersed in fluorescein solution, with a single μLED activated (*blue*), showing fluorescence (*green*) only beyond the tip of the lit needle. There is some weak leakage through the base of the shank, but no discernable coupling into, or emission from, adjacent needles. This image indicates slight misalignment between the μLED and optical vias, but the fact that this misalignment does not introduce cross-talk into adjacent needles demonstrates the effectiveness of the interposer design. The needle tips of this UOA device are blunter that in the device used in the *in vivo* studies.

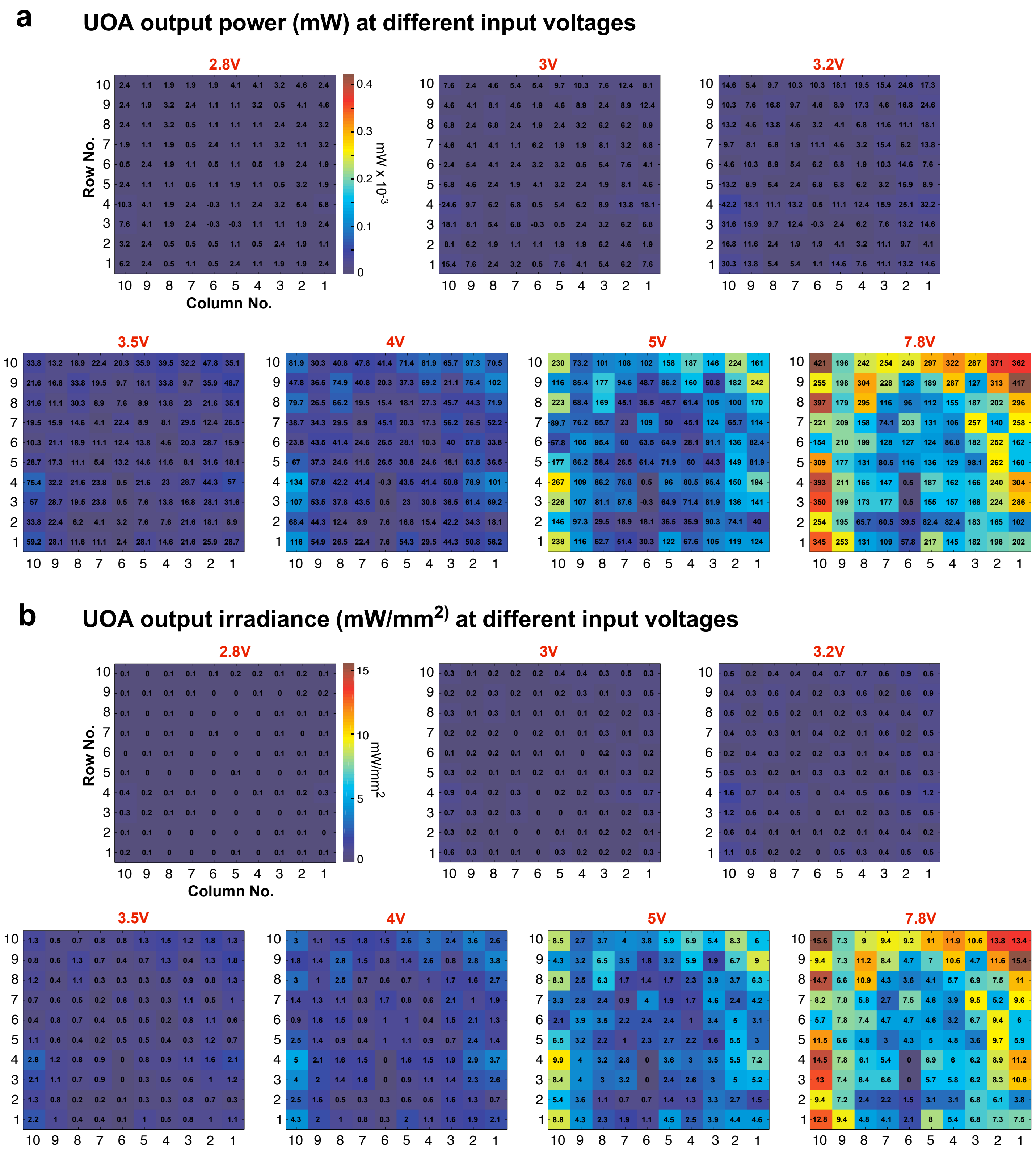

Supplementary Figure 2

Output optical power and irradiance of the UOA

Each heat map represents the output optical power **(a)** or irradiance **(b)** measured at each needle tip across the entire UOA for different input voltages (indicated at the top of each map). We defined the irradiance as the emitted optical power divided by the area of the emission surface. Needles C6-R3 and C6-R4 did not emit light at their tips, thus, they were not included in the descriptive statistics in Supplementary Table 1.

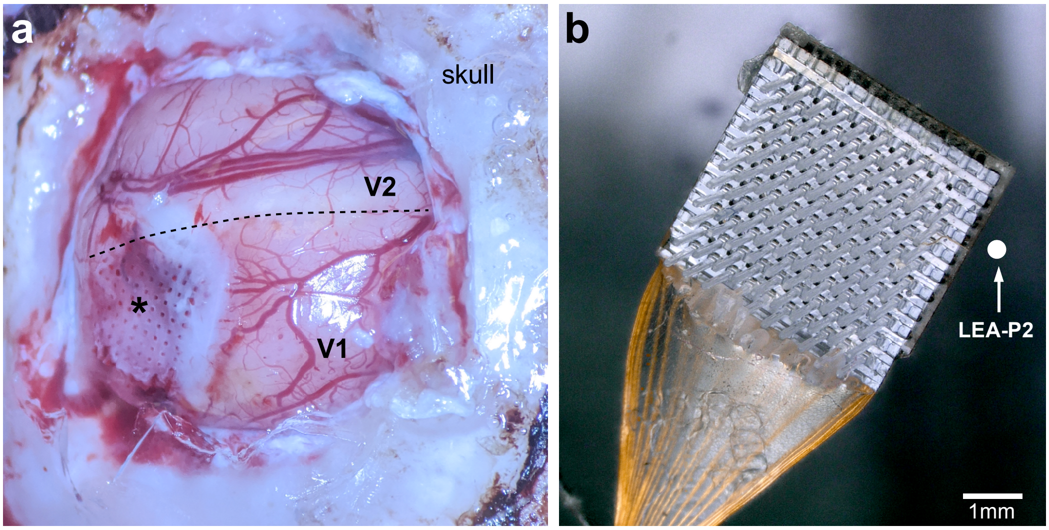

Supplementary Figure 3

UOA after explantation
(a) Image of the brain after explantation of the UOA at the end of the *in vivo* testing experiment. The *asterisk* marks the center of the UOA implantation site. The white area at the site of the explantation is the *Duragel* that was placed on the brain after insertion of the UOA to protect the cortical surface and prevent deydration. The *dashed line* marks the border between V2 and V1. **(b)** The UOA after explantation. The *white dot* indicates the approximate location of the LEA used for penetration 2 (P2) relative to the UOA. The shank at the top right corner (column 1 row 10) was broken prior to the UOA insertion. The remaining shanks are intact.

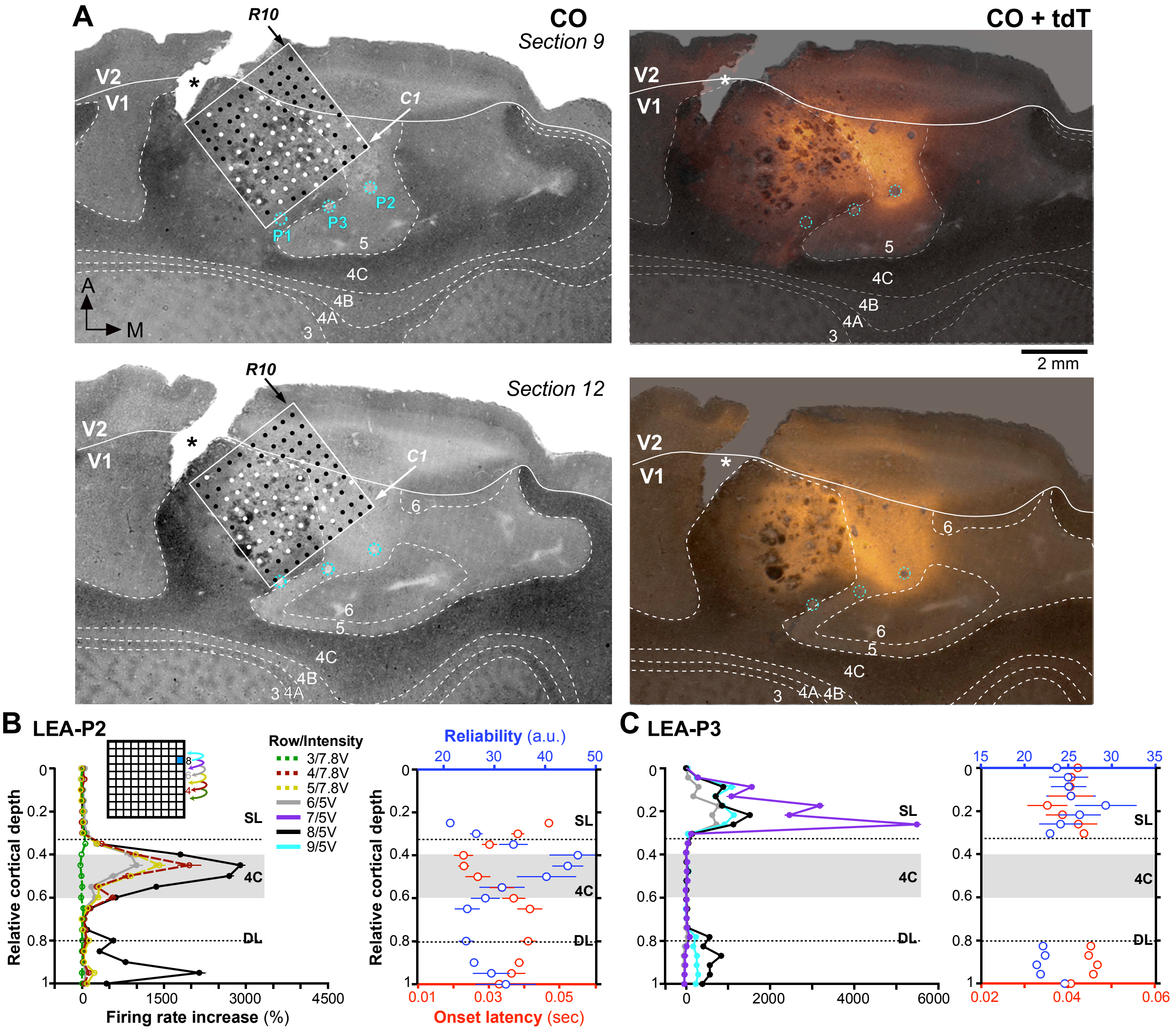

Supplementary Figure 4

Laminar and tangential location of UOA and LEA penetrations

(a) **Left:** The locations of the UOA (*white box*) and of 3 LEA penetrations *(P1-P3, cyan dashed circles*) are shown on two cytochrome-oxidase (CO)-stained tangential sections through V1 and V2 (top section is more superficial). *Solid white contour*: V1/V2 border; *dashed white contours* delineate V1 layers (indicated). The location of column 1 (*C1*) and row 10 (*R10*) are indicated by *arrows*. *White dots* mark the locations of UOA needles visible in these sections as cortical damage. *Black dots* mark the location of UOA needles visible in more superficial sections, but not in these sections. Note that the majority of white dots are located in layer 4C in both sections. There are no white dots in column 1 in section #12 and only 2 in section #9, as the needle tips in this column terminated in the sections just above, thus in the superficial part of L4C. The postero-lateral half of the UOA terminated in slightly more superficial layers compared to its antero-medial half. *A:*anterior; *M:* medial. **Right:** Same CO-stained section as shown on the left, with superimposed image of the same section viewed under tdT fluorescence. The fluorescent image was rendered transparent in Adobe Photoshop. P2 and P3 were located inside or near, respectively, the region of tdT/ChR2 expression, whereas P1 was more distant from it; accordingly, only the neurons recorded in P2 and P3, but not in P1, could be modulated by the UOA. P2 was located about 1-1.1 mm medial to the nearest UOA needle (C1-R8, C1-R9, in these sections), while P3 was located about 800µm from the UOA (C1-R5 and C1-R4 are the nearest needles to P3 in these sections, but, as this penetration was not vertical, the more superficial LEA contacts were closer to needles C1-R6 and C1-R7 and more distant from the UOA, as also indicated by the physiological recordings in (c), and in **Fig. 3f**). The *asterisk* in all panels marks a crack in the tissue caused by histological processing, not by the UOA insertion. **(b)** **Left:** Relative cortical depth of each contact on the LEA in P2 is plotted versus the increase in firing rate caused by stimulation of single µLEDs along column 1 (*inset*). Different color traces are data for different µLEDs (rows 3-9) at 5 or 7.8V stimulation intensity (the most distant µLEDs only evoked responses at the higher intensity). µLED C1-R8 evoked the max response, indicating this needle tip was the closest to P2. **Right:** Relative cortical depth on the LEA-P2 is plotted versus the mean onset latency (*red*) or the mean onset latency reliability (*blue*; inverse of the SD of the distribution of pulse by pulse onset latencies) of responses at each contact evoked by stimulation of the whole µLED array; means are averages across all photostimulation intensities ≤5V (n= 160-255 pulses/condition). The shortest and most reliable response latencies are for contacts in L4C, indicating the UOA tips nearest P2 ended in this layer. **(c)** Same as in (b) but for P3 (n=125-185 pulses/condition). The data indicate that the µLED closest to P3 was C1-R7 whose tip terminated in the superficial layers. Error bars: s.e.m.

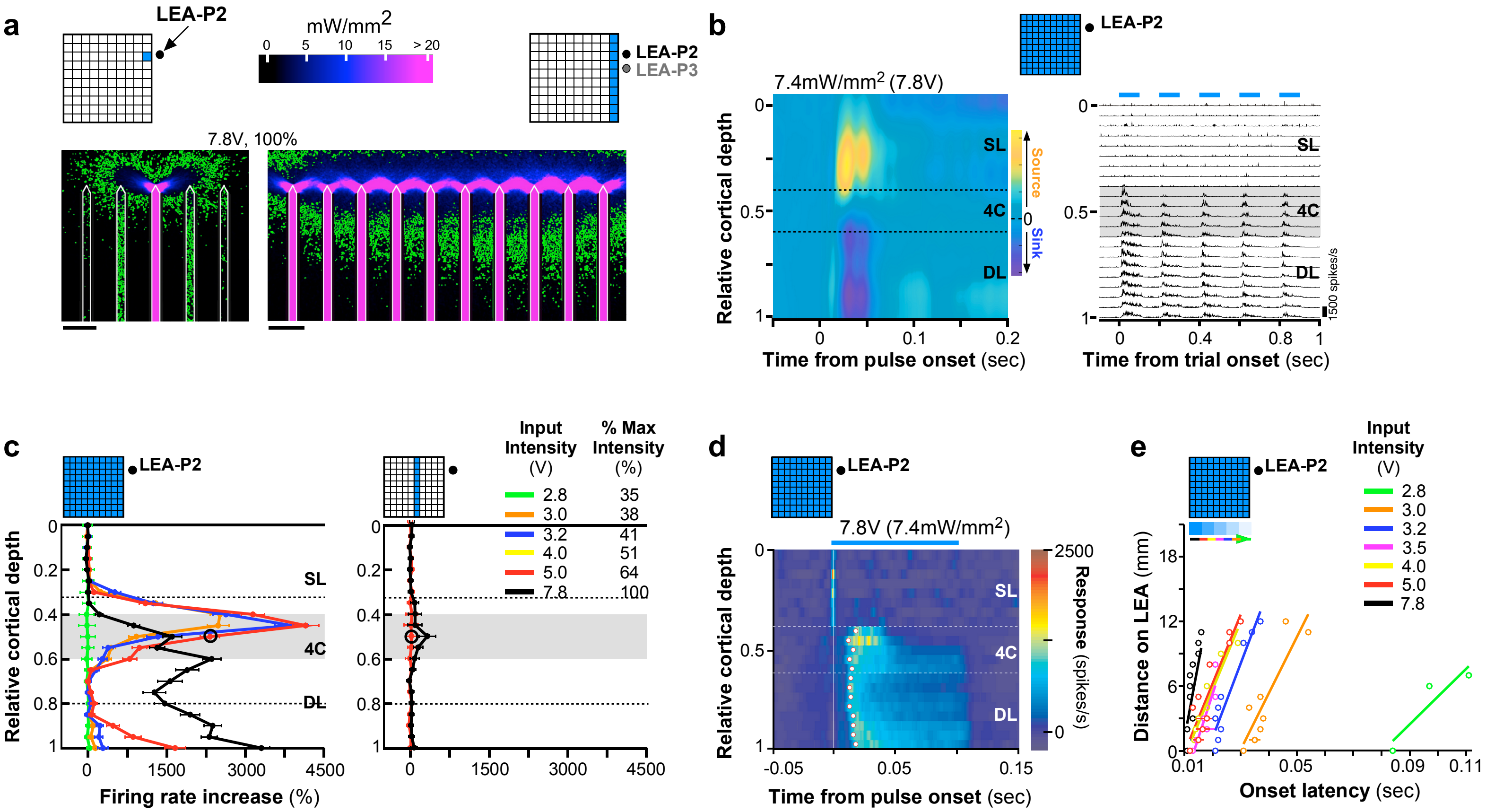

Supplementary Figure 5

Bench testing and *in vivo* results for UOA stimulation intensity of 7.8V

(a) **Left:** Ray trace model of light spread in cortical tissue when a single µLED (C1R8, i.e. the closest to LEA-P2) is activated at 7.8V. **Right:** Model of light spread in tissue when all of column 1 (nearest to both LEA-P2 and P3) is activated at 7.8V. Green contour encloses tissue volume within which the light irradiance is above 1mW/mm^2. Scale bars: 400µm. **(b)** CSD analysis (**Left**) and MUA (**Right**) through the depth of V1 for LEA-P2 in response to phasic UOA photostimulation (100ms pulse, 5Hz, 7.4mW/mm^2; n=155 pulses) of the entire UOA. Conventions are as in **Fig. 2d**. Current sinks and strong phasic MUA in response to UOA stimulation extended from L4C to the white matter boundary. **(c)** Relative cortical depth of contacts on LEA-P2 plotted versus firing rate increase in response to stimulation of the whole UOA (Left; n=25-205 pulses/condition) or Column 5 (Right; n=155-160 pulses/condition) at different intensities (different color traces). Same plots as shown in **Fig. 2f and i**, respectively, with added data from 7.8V stimulation. **Left:** Relative to stimulation at lower intensities, 7.8V stimulation of the whole UOA resulted in a decreased activation peak in L4C and increased responses in L5-6. At this higher intensity, light spread through the cortical depth may have directly contributed to firing rate increase in L5 and 6, while the reduced peak in L4C may have resulted from activation of higher threshold inhibitory networks. **Right:** Stimulation of Column 5 evoked a response in L4C only at the highest intensity used (7.8V). **(d)** Heatmap of MUA through the depth of V1 during the peri-pulse period, when the whole UOA was stimulated at 7.8V (n=155 pulses). Other conventions are as in **Fig. 4**. There was no difference in onset latency of evoked MUA across layers suggesting light spreading deep into tissue at this intensity directly activated both mid- and deep layers. **(e)** Distance on the LEA of each contact from the contact with the fastest onset latency plotted against onset latency, for the whole UOA condition. Same as in **Fig. 5a** with added data from 7.8V stimulation (n=125-185 pulses). Error bars in all panels: s.e.m.

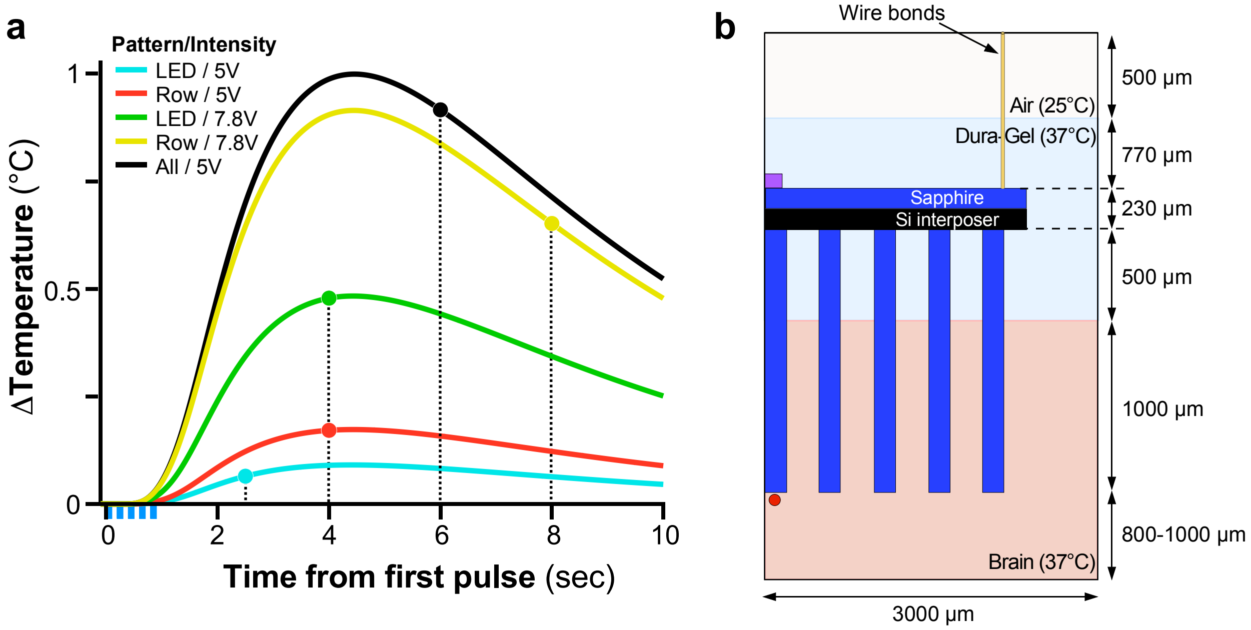

c

Material	Thermal Conductivity (W/m/°C)	Heat Capacity (J/kg/°C)	Density (kg/m^3)
Brain	0.55	3700	1040
Dura-Gel	0.2	840	970
Silicon	148	710	2330
Sapphire	40	700	3980
Glass needle	1	800	2500
Gold (wire bonds)	301	128	19320

Supplementary Figure 6

Simulated temperature near the UOA tips (in L4C) under different photostimulation conditions. (a) Simulated temperature time profile in tissue under different operating conditions of the UOA, including a single µLED (*LED*), an entire row of µLEDs (*Row*), or the entire UOA (*All*), under different light intensities (5V or 7.8V). Heat generation occurs on the topside of the device and, due to the intervening Dura-Gel layer and tissue (superficial cortical layers 1-3), there is a delay in temperature rise at the stimulation site in L4 (*red dot* in panel b). This delay is approximately equal to the optical pulse stimulation period (five pulses at 100ms on and 100ms off, denoted as *blue bars* below the abscissa; the last optical pulse turns off at 0.9 sec). Temperature continues to rise during the first few seconds of the inter-trial interval (ITI) (the end of each ITI is marked by *colored dots;* longer ITIs were used for larger area and higher stimulation intensities), and peaks at ≤1°C for all conditions. Pulse pile-up is observed in certain conditions, resulting in a maximum temperature 31% ± 1% higher than the peak temperature observed after the initial pulse. **(b)** Illustration of the simulation geometry, showing the UOA glass needles, Si interposer layer, Sapphire substrate, and µLED (*purple*, only one illustrated). Gold wire bonds were also included (only one illustrated). Since the device was only partially inserted, the non-inserted portion is embedded in Dura-Gel. The air region was held at 25°C, the right-most Dura-Gel and brain boundaries were held at 37°C, and the lower brain boundary was held at 37°C (essential boundary conditions). A rotational symmetry boundary condition was used along the left edge. **(c)** Relevant properties of the constituent materials. A perfusion rate of 60 ml/min per 100g of tissue was included for brain.

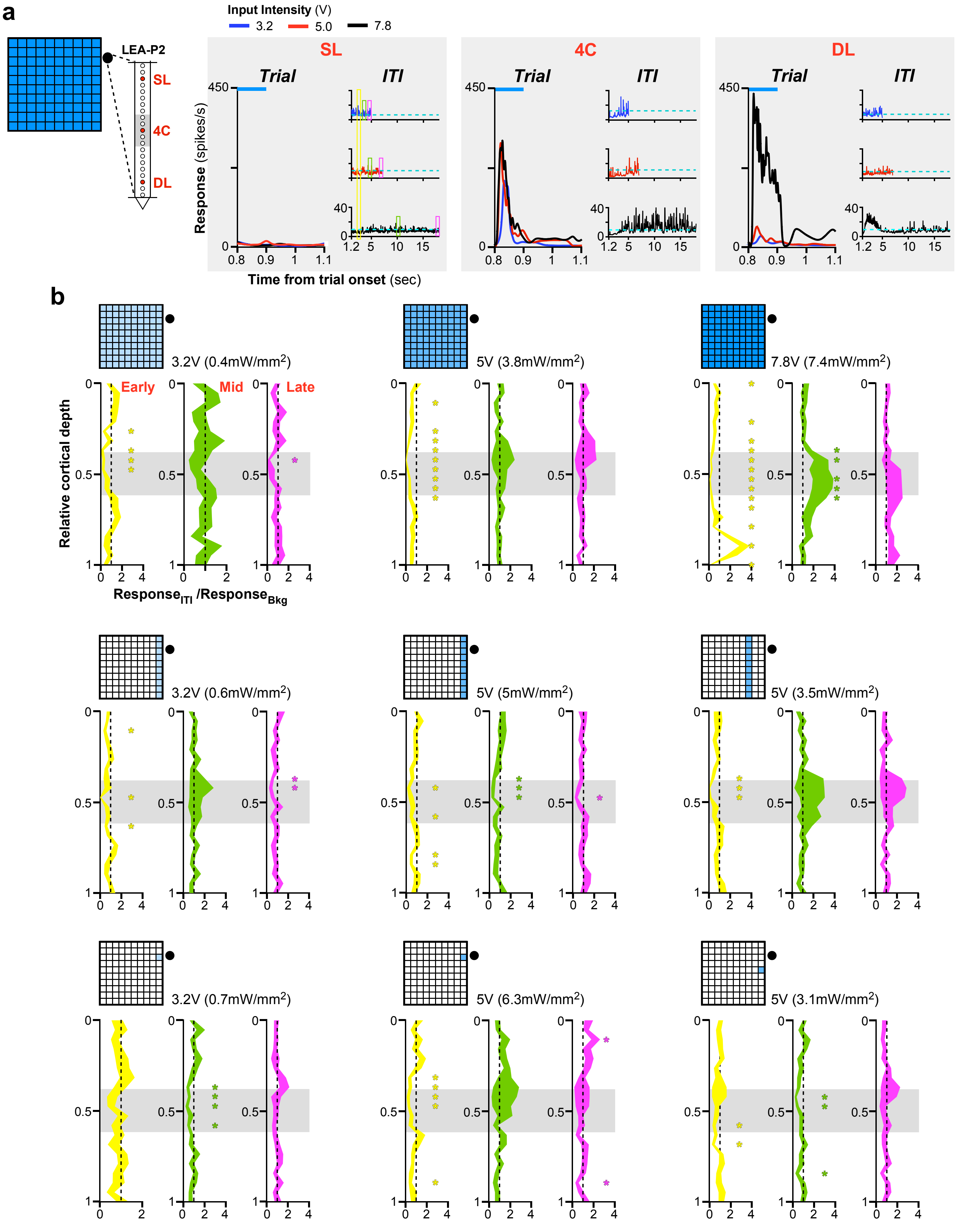

Supplementary Figure 7

Neuronal activity during the inter-trial period.

(a) **Left**: Schematics of whole array UOA stimulation and of LEA in P2. Data from the 3 highlighted LEA contacts in superficial layers (SL), L4C, and deep layers (DL), respectively, are shown on the right. **Right**: In each panel, trial-aligned PSTHs for the last portion of the trial period (**Left**) and the inter-trial interval (ITI; **Right**) are shown for the highlighted channels at three intensities of whole array stimulation. Each trial consisted of 1sec of photostimulation (100msec pulse duration, 5Hz; n=125-155 pulses/condition) followed by 1.5-21sec ITI. *Blue bar*: final 100ms stimulus pulse period. The *yellow* (early), *green* (middle), and *magenta* (late) *boxes* in the ITI plots for the example SL channel indicate the portions of the ITI that were compared with the average ongoing activity on each channel prior to the beginning of the experiment ("background"). *Dashed cyan lines*: average MUA spike rate during a brief background period prior to each experiment. For the example contacts, there was an increase in MUA activity, relative to the average background rate, during the ITI for the 7.8V intensity in L4C and the early ITI period in the DL. **(b)** Comparison of early, middle, and late ITI period activity with ongoing background activity. For each condition shown in **Fig. 4c**, for all LEA contacts in V1, we compared MUA spike rates during the indicated early, middle, and late portions of the ITI period with the average MUA spike rate over the "background" period. UOA-stimulation conditions are shown schematically at the top left of each panel. Data are plotted as relative cortical depth versus average early (**Left**), middle (**Center**), or late (**Right**) ITI response normalized to the average background response (shaded regions: s.e.m.; n=125-205 pulses/condition). *Asterisks* indicate significant differences in ITI and background response (t-test). Across all, but the whole array/7.8V condition, channels that responded to UOA stimulation (cf. **Fig. 2d-i and Fig.4c**) tended to show a significant decrease in MUA spiking, relative to the average background rate, particularly in the early portion of the ITI. Only few contacts showed significant changes in the later portion of the ITI and for all, but one, the changes were decreases in MUA spiking relative to background. The one exception to this pattern was seen on the responsive (deep-) mid-layer contacts during whole array/7.8V stimulation. On these contacts, there was a sharp increase in activity relative to baseline in the (early) middle period of the ITI. However, there was no significant difference between late ITI activity and background activity during this condition, indicating that by the end of the long ITI period spiking activity had returned to baseline. Importantly, these results indicate that the increases in MUA activity and their specific laminar patterns induced by UOA activation cannot be explained by thermal artifacts (see also **Supplementary Fig. 6**).

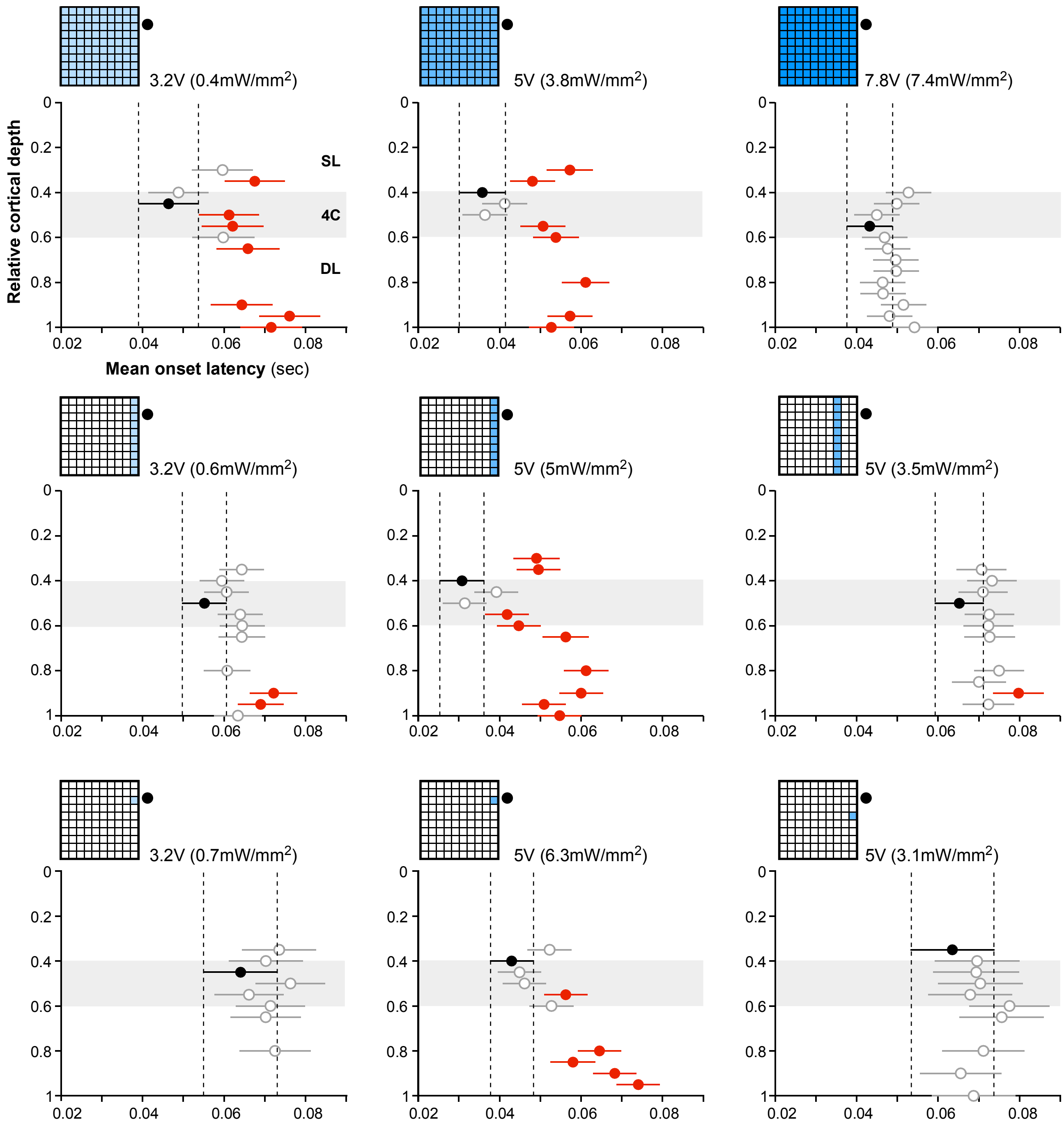

Supplementary Figure 8

Statistical analysis of onset latencies for penetration 2 (data shown in Figure 4c)
Mean onset latency (± s.e.m; n=125-205 pulses/condition) for each contact in LEA-P2 which showed significant response to UOA stimulation, for the UOA stimulation condition indicated by the *insets* at the top left of each plot. The mean latency was estimated from distributions of single-trial latency estimates. The *black dot* indicates the contact with the shortest latency in each condition. The *red dots* indicate the contacts that showed a statistically significant (Tukey HSD test) pairwise difference with the shortest latency contact (*black dot*), and the *empty dots* the contacts that did not differ significantly from the black dot. The *vertical dashed lines* indicate the points beyond which comparisons are significant.

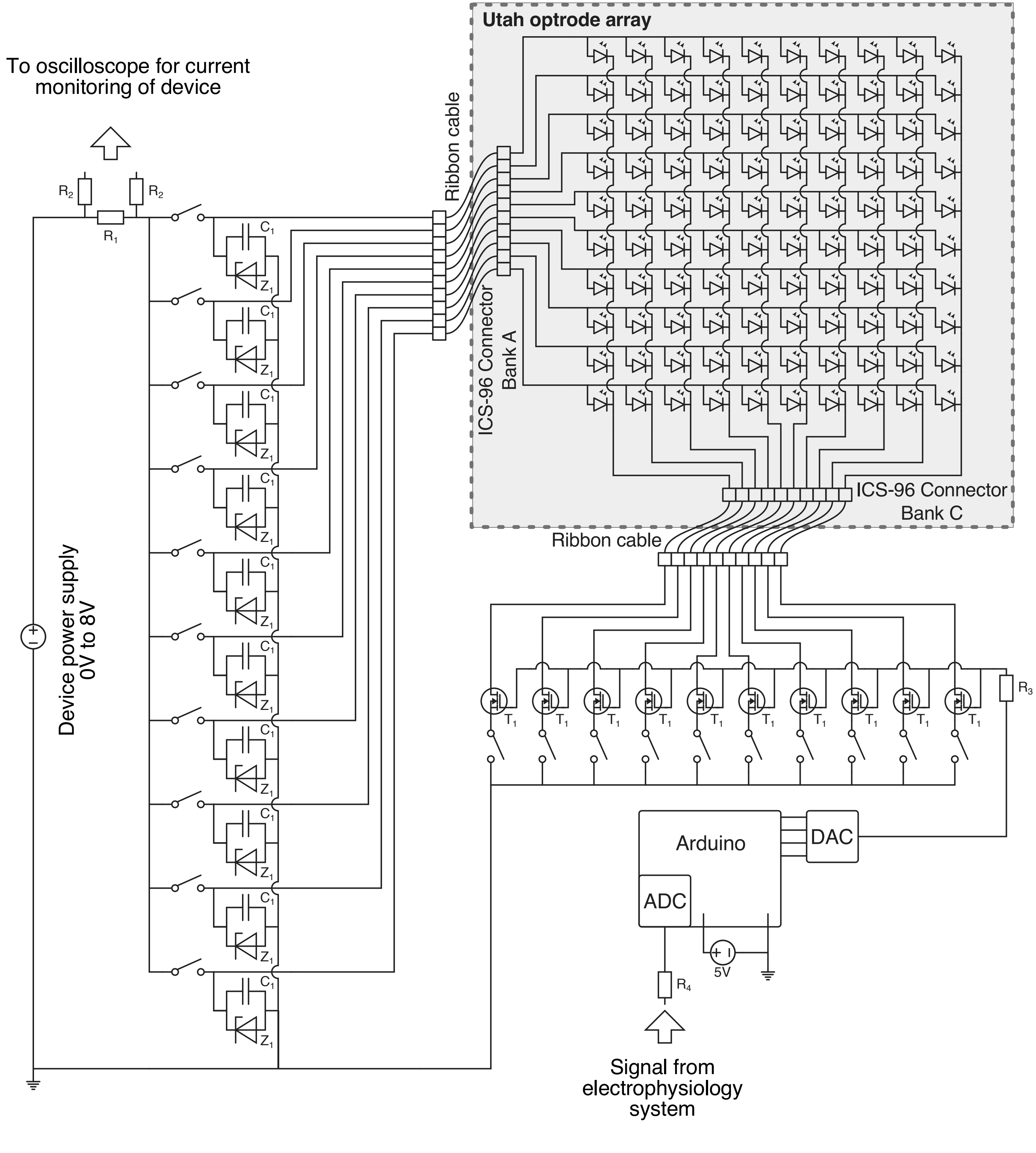

Supplementary Figure 9

Circuit Diagram of the UOA Driver Board

For the experiments described in the main manuscript, the following parameters for the circuit components were: R_1) 0.2 Ω (assembled from five parallel parallel-connected 1 Ω, 1 W resistors to ensure functionality at higher currents); C_1) I μF; Z_1) 8.2 V breakdown voltage, 1 W; T_1) TN0606 low-threshold n-channel enhancement-mode MOSFET, 60V, 500 mA; R_2)2 MΩ; R_3)301 Ω; R_4)51 Ω. The intent for the design was to ensure simplicity and ease-of-use across labs. The UOA is connected to the driver via ribbon cables. To choose the illumination pattern, 20 manual mechanical switches (10 for the rows and 10 for the columns of the 10x10 matrix addressed UOA) are used. The array itself is powered by a lab DC power supply that is set manually to the desired voltage. On the positive voltage side, a test resistor (R_1) is introduced after the power source to monitor the current going through the device (by observing the voltage drop over the test resistor on an oscilloscope). Here, the electric path is split to the switches selecting the active rows on the device. The capacitor (C_1) and Zener diode (Z_1) arrangements on the positive voltage side after the switches are intended to dampen potential voltage spikes originating from switch operation while the device is powered on. On the negative voltage side, besides the mechanical switches to select the active columns of the device, enhancement mode MOSFETs (T_1) are introduced to translate the voltage pulse trains of the trigger signal into temporal activation patterns of the μLEDs. It was found that directly applying a square wave trigger signal to the transistors could lead to capacitive voltage artifacts. Thus, the trigger signal is first processed via an Arduino with a digital-to-analog converter (DAC). Here, the signal is picked up by the Arduino's internal analog-to-digital converter and then translated via the connected DAC to a signal that includes a linear voltage ramp. When applied to the transistors, this new signal, in combination with the transistor's current-to-gate-source-voltage characteristic, ensures smoothly rising and falling voltage flanks of the operating voltage (and thus prevents measurable voltage artifacts). The remaining resistors (R_{2}-$_{4}$) protect the measurement equipment, Arduino and transistors, respectively. Please note that if electrical control of the illumination pattern is desired, the manual switches could be replaced by relays (for example, externally controlled by Arduinos) or by transistors. For more complex applications, the electronics can be easily redeveloped, as the UOA is based on a straightforward matrix-addressed 10x10-μLED array.

Supplementary Table 1

Measured Mean Output Photostimulation Intensities for Different Input Voltages

WHOLE ARRAY

Input Voltage (V)	Output Optical Power (mW)					
	Mean	*SD*	*Median*	*Min*	*Max*	*IQR*
2.8	0.0022	0.0016	0.0019	0	0.010	0.0014
3	0.0057	0.0040	0.0050	0.0005	0.024	0.0051
3.2	0.011	0.0072	0.010	0.0010	0.042	0.0091
3.5	0.022	0.013	0.020	0.0024	0.075	0.0170
4	0.044	0.026	0.041	0.0075	0.13	0.0313
5	0.10	0.056	0.088	0.018	0.27	0.0629
7.8	0.19	0.09	0.18	0.039	0.42	0.12

Input Voltage (V)	Output Irradiance (mW/mm^2)					
	Mean	*SD*	*Median*	*Min*	*Max*	*IQR*
2.8	0.08	0.06	0.07	0.01	0.38	0.05
3	0.21	0.14	0.20	0.02	0.91	0.19
3.2	0.41	0.27	0.38	0.04	1.56	0.33
3.5	0.82	0.49	0.75	0.09	2.79	0.62
4	1.67	0.95	1.53	0.28	4.98	1.16
5	3.79	2.08	3.33	0.67	9.88	2.48
7.8	7.4	3.19	6.9	1.45	15.6	4.46

COLUMN 1

Input Voltage (V)	Output Optical Power (mW)					
	Mean	*SD*	*Median*	*Min*	*Max*	*IQR*
2.8	0.0030	0.0016	0.0024	0.0010	0.0068	0.0013
3	0.0079	0.0046	0.0071	0.0019	0.0181	0.0043
3.2	0.0156	0.0082	0.0146	0.0040	0.0321	0.0091
3.5	0.0305	0.0146	0.0301	0.0089	0.0570	0.0170
4	0.0611	0.0276	0.0627	0.0181	0.1016	0.0354
5	0.1350	0.0597	0.1324	0.0400	0.2424	0.0873
7.8	0.2548	0.0981	0.2719	0.1016	0.4168	0.1411

Input Voltage (V)	Output Irradiance (mW/mm^2)					
	Mean	*SD*	*Median*	*Min*	*Max*	*IQR*

2.8	0.11	0.06	0.09	0.04	0.25	0.05
3	0.29	0.17	0.27	0.07	0.67	0.16
3.2	0.58	0.31	0.54	0.15	1.19	0.34
3.5	1.13	0.54	1.12	0.33	2.11	0.63
4	2.26	1.02	2.32	0.67	3.77	1.31
5	5.00	2.21	4.90	1.48	8.98	3.23
7.8	9.43	3.63	10.07	3.77	15.44	5.23

COLUMN 3

**Input Voltage** (V)	**Output Optical Power** (mW)					
	Mean	***SD***	***Median***	***Min***	***Max***	***IQR***
2.8	0.0020	0.0010	0.0022	0.0005	0.0032	0.0021
3	0.0055	0.0023	0.0058	0.0019	0.0089	0.0043
3.2	0.0106	0.0044	0.0110	0.0032	0.0159	0.0078
3.5	0.0211	0.0080	0.0216	0.0081	0.0321	0.0119
4	0.0421	0.0145	0.0432	0.0181	0.0657	0.0143
5	0.0933	0.0303	0.0932	0.0443	0.1460	0.0232
7.8	0.1836	0.0550	0.1820	0.0981	0.2871	0.0202

**Input Voltage** (V)	**Output Irradiance** (mW/mm^2)					
	Mean	***SD***	***Median***	***Min***	***Max***	***IQR***
2.8	0.07	0.04	0.08	0.02	0.12	0.08
3	0.20	0.09	0.22	0.07	0.33	0.16
3.2	0.39	0.16	0.41	0.12	0.59	0.29
3.5	0.78	0.30	0.80	0.30	1.19	0.44
4	156	0.54	1.60	0.67	2.43	0.53
5	3.46	1.12	3.46	1.64	5.40	0.86
7.8	6.80	2.03	6.74	3.63	10.63	0.75

COLUMN 5

**Input Voltage** (V)	**Output Optical Power** (mW)					
	Mean	***SD***	***Median***	***Min***	***Max***	***IQR***
2.8	0.0014	0.0011	0.0010	0.0003	0.0041	0.0008
3	0.0041	0.0028	0.0032	0.0005	0.0097	0.0035
3.2	0.0081	0.0051	0.0068	0.0024	0.0181	0.0070
3.5	0.0165	0.0110	0.0142	0.0076	0.0360	0.0127
4	0.0344	0.0177	0.0249	0.0168	0.0713	0.0232
5	0.0797	0.0376	0.0683	0.0365	0.1583	0.0460
7.8	0.1623	0.0620	0.1456	0.0824	0.2971	0.0651

Input Voltage (V)	Output Irradiance (mW/mm^2)					
	Mean	***SD***	***Median***	***Min***	***Max***	***IQR***
2.8	0.05	0.04	0.04	0.01	0.15	0.03
3	0.15	0.11	0.12	0.02	0.36	0.13
3.2	0.30	0.18	0.25	0.09	0.67	0.26
3.5	0.61	0.36	0.53	0.28	1.33	0.47
4	1.27	0.66	1.09	0.62	2.64	0.86
5	2.95	1.39	2.53	1.35	5.87	1.7
7.8	6.03	2.30	5.39	3.05	11.00	2.41

SINGLE µLEDs IN COLUMN 1

Input Voltage (V)	Mean Output Optical Power (mW)								
	Row 1	***Row 2***	***Row 3***	***Row 4***	***Row 5***	***Row 6***	***Row 7***	***Row 8***	***Row 9***
2.8	0.0024	0.0011	0.0024	0.0068	0.0019	0.0019	0.0032	0.0032	0.0046
3	0.0076	0.0019	0.0068	0.0181	0.0046	0.0041	0.0068	0.0089	0.0124
3.2	0.0146	0.0040	0.0146	0.0322	0.0089	0.0076	0.0138	0.0181	0.0246
3.5	0.0287	0.0089	0.0316	0.0570	0.0181	0.0159	0.0265	0.0351	0.0487
4	0.0562	0.0181	0.0691	0.1010	0.0365	0.0338	0.0521	0.0719	0.1016
5	0.1243	0.0400	0.1406	0.1938	0.0819	0.0824	0.1138	0.1697	0.2425
7.8	0.2016	0.1016	0.2857	0.3035	0.1597	0.1624	0.2581	0.2957	0.4168

Input Voltage (V)	Mean Output Irradiance (mW/mm^2)								
	Row 1	***Row 2***	***Row 3***	***Row 4***	***Row 5***	***Row 6***	***Row 7***	***Row 8***	***Row 9***
2.8	0.1	0	0.1	0.3	0.1	0.1	0.1	0.1	0.2
3	0.3	0.1	0.3	0.7	0.2	0.2	0.3	0.3	0.5
3.2	0.5	0.2	0.5	1.2	0.3	0.3	0.5	0.7	0.9
3.5	1.1	0.3	1.2	2.1	0.7	0.6	1	1.3	1.8
4	2.1	0.7	2.6	3.7	1.4	1.3	1.9	2.7	3.8
5	4.6	1.5	5.2	7.2	3	3.1	4.2	6.3	9
7.8	7.5	3.8	10.6	11.2	5.9	6	9.6	11	15.4
