# Supplementary material for: An optrode array for spatiotemporally-precise large-scale optogenetic stimulation of deep cortical layers in non-human primates
Source: Commun Biol. 2024 Mar 14;7:329. doi: 10.1038/s42003-024-05984-2 (PMC10940688; doi:10.1038/s42003-024-05984-2)
Supplement: Supplementary file 3 — Description of Additional Supplementary Files [file 42003_2024_5984_MOESM3_ESM.pdf]

Description of Additional Supplementary Files

File name: Supplementary Data

Description: Source data file for figures and Supplementary figures.
