# Supplementary material for: An optrode array for spatiotemporally-precise large-scale optogenetic stimulation of deep cortical layers in non-human primates
Source: Commun Biol. 2024 Mar 14;7:329. doi: 10.1038/s42003-024-05984-2 (PMC10940688; doi:10.1038/s42003-024-05984-2)
Supplement: Supplementary file 5 — Reporting Summary [file 42003_2024_5984_MOESM5_ESM.pdf]

Corresponding author(s): Angelucci/Blair
Last updated by author(s): Feb 5, 2024

Reporting Summary

Nature Portfolio wishes to improve the reproducibility of the work that we publish. This form provides structure for consistency and transparency in reporting. For further information on Nature Portfolio policies, see our Editorial Policies and the Editorial Policy Checklist.

Statistics

For all statistical analyses, confirm that the following items are present in the figure legend, table legend, main text, or Methods section.

n/a	Confirmed	
☐	☒	The exact sample size (n) for each experimental group/condition, given as a discrete number and unit of measurement
☐	☒	A statement on whether measurements were taken from distinct samples or whether the same sample was measured repeatedly
☐	☒	The statistical test(s) used AND whether they are one- or two-sided<br>*Only common tests should be described solely by name; describe more complex techniques in the Methods section.*
☐	☒	A description of all covariates tested
☐	☒	A description of any assumptions or corrections, such as tests of normality and adjustment for multiple comparisons
☐	☒	A full description of the statistical parameters including central tendency (e.g. means) or other basic estimates (e.g. regression coefficient) AND variation (e.g. standard deviation) or associated estimates of uncertainty (e.g. confidence intervals)
☐	☒	For null hypothesis testing, the test statistic (e.g. F, t, r) with confidence intervals, effect sizes, degrees of freedom and P value noted<br>*Give P values as exact values whenever suitable.*
☒	☐	For Bayesian analysis, information on the choice of priors and Markov chain Monte Carlo settings
☒	☐	For hierarchical and complex designs, identification of the appropriate level for tests and full reporting of outcomes
☒	☐	Estimates of effect sizes (e.g. Cohen's d, Pearson's r), indicating how they were calculated

Our web collection on statistics for biologists contains articles on many of the points above.

Software and code

Policy information about availability of computer code

Data collection	Central Software Suite Version 7.5.2 (Blackrock Neurotech, Salt Lake City, UT, USA) - e-phys data acquisition<br>Zen (blue edition) Version 2.6 (Carl Zeiss Microscopy, GmbH, DE) - histological image acquisition
Data analysis	NPMK toolbox (Blackrock Neurotech, Salt Lake City, UT, USA - https://github.com/BlackrockNeurotech/NPMK/releases_ - Matlab functions for loading e-phys data<br>Chronux toolbox (http://chronux.org) - Matlab functions for analyzing e-phys data<br>No novel or non-standard methodology was used in the data analysis reported in this manuscript. All functions used for analysis of e-phys data were standard implementations in MATLAB 2017a (Mathworks Inc., Natick, MA, USA)<br><br>Histological Analyses were performed with:<br>Neurolucida Version 11.09 (Microbrightfield Bioscience, VT, USA)<br>Adobe Photoshop Version 6 (Adobe Inc., Salt Lake City, USA)

For manuscripts utilizing custom algorithms or software that are central to the research but not yet described in published literature, software must be made available to editors and reviewers. We strongly encourage code deposition in a community repository (e.g. GitHub). See the Nature Portfolio guidelines for submitting code & software for further information.

Data

Policy information about availability of data

All manuscripts must include a data availability statement. This statement should provide the following information, where applicable:

- Accession codes, unique identifiers, or web links for publicly available datasets
- A description of any restrictions on data availability
- For clinical datasets or third party data, please ensure that the statement adheres to our policy

The data that supports the findings of this study are available from the corresponding authors upon reasonable request. Source data for the figures are provided with the paper

Human research participants

Policy information about studies involving human research participants and Sex and Gender in Research.

Reporting on sex and gender	no human subjects
Population characteristics	*Describe the covariate-relevant population characteristics of the human research participants (e.g. age, genotypic information, past and current diagnosis and treatment categories). If you filled out the behavioural & social sciences study design questions and have nothing to add here, write "See above."*
Recruitment	*Describe how participants were recruited. Outline any potential self-selection bias or other biases that may be present and how these are likely to impact results.*
Ethics oversight	*Identify the organization(s) that approved the study protocol.*

Note that full information on the approval of the study protocol must also be provided in the manuscript.

Field-specific reporting

Please select the one below that is the best fit for your research. If you are not sure, read the appropriate sections before making your selection.

☒ Life sciences ☐ Behavioural & social sciences ☐ Ecological, evolutionary & environmental sciences

For a reference copy of the document with all sections, see nature.com/documents/nr-reporting-summary-flat.pdf

Life sciences study design

All studies must disclose on these points even when the disclosure is negative.

Sample size	A sample size of at least two animals is standard methodology in non-human primate systems neuroscience (cf. Maris & Fries (2021). arXiv). We used three animals in the current study.
Data exclusions	We excluded one LEA penetration from the analysis as the electrode was placed in a region too far from ChR2 expression/key UOA sites to evoke measurable responses at any intensity
Replication	We performed at least one independent replication in both the electrophysiology and c-Fos studies.
Randomization	Not relevant because all animals received the same treatment
Blinding	Not relevant because all animals received the same treatment

Reporting for specific materials, systems and methods

We require information from authors about some types of materials, experimental systems and methods used in many studies. Here, indicate whether each material, system or method listed is relevant to your study. If you are not sure if a list item applies to your research, read the appropriate section before selecting a response.

Materials & experimental systems

n/a	Involved in the study	
☐	☒	Antibodies
☒	☐	Eukaryotic cell lines
☒	☐	Palaeontology and archaeology
☐	☒	Animals and other organisms
☒	☐	Clinical data
☒	☐	Dual use research of concern

Methods

n/a	Involved in the study	
☒	☐	ChIP-seq
☒	☐	Flow cytometry
☒	☐	MRI-based neuroimaging

Antibodies

Antibodies used	-Rabbit anti-c-Fos (Abcam, MA, USA), Catalogue No. 190289<br>-Donkey anti-rabbit IgG-AF647 (Jackson ImmunoResearch, PA, USA), Catalogue No. A-31573
Validation	-Rabbit anti-c-Fos (paraphrased from manufacturer website): Immunohistochemistry - Free floating analysis of mouse hippocampus or olfactory bulb sections that were labeled with ab190289 at 1/20000. Following transcardial perfusion of mouse with 4% paraformaldehyde, brain was post-fixed for 24 hours, cut to 45 microns, and free-floating sections were stained with ab190289 at above dilution. The c-Fos antibody (ab190289) stains only the nuclei of spontaneously active neurons.

Animals and other research organisms

Policy information about studies involving animals; ARRIVE guidelines recommended for reporting animal research, and Sex and Gender in Research

Laboratory animals	Macaca fasicularis, three females between the ages of 2-5 years
Wild animals	The study did not include wild animals.
Reporting on sex	The study only included female animals.
Field-collected samples	The study did not include field collected samples.
Ethics oversight	University of Utah Institutional Animal Care and Use Committee

Note that full information on the approval of the study protocol must also be provided in the manuscript.
